# Design and construction of a low-cost, low-input Open Top Chamber field warming setup to assess aboveground plant response to global warming

**DOI:** 10.3389/fpls.2025.1677291

**Published:** 2025-10-14

**Authors:** Joshua Hauser, Pieter C. Kooijman, Edward N.D. Paddon, Ava Verhoeven, Jasmijn Kalisvaart, Adam N. Meesters, Basten L. Snoek, Martijn van Zanten

**Affiliations:** ^1^ Plant Stress Resilience, Institute of Environmental Biology, Faculty of Sciences, Utrecht University, Utrecht, Netherlands; ^2^ Lili’s Proto Lab, Faculty of Sciences, Utrecht University, Utrecht, Netherlands; ^3^ Theoretical Biology & Bioinformatics, Institute of Biodynamics and Biocomplexity, Utrecht University, Utrecht, Netherlands

**Keywords:** global warming, field warming, open top chambers, arabidopsis, tomato, snowdrops, *Galanthus nivalis*

## Abstract

Climate change drastically impacts the development, physiology, and phenology of plants. Conducting experiments to elucidate plant responses to high temperatures is essential to understanding and mitigating the impact of global warming. Typically, empirical research assessing the impact of (high) temperatures is conducted in climate-controlled growth chambers, cabinets, or greenhouses. Although informative, such experiments ignore the effects that seasonal, daily, and minute-scale changes in environmental parameters can have on temperature responsiveness. Semi-controlled field warming setups are therefore required in which average temperatures are consistently raised whereas other environmental parameters, such as diurnal fluctuations in temperature, rainfall, changes in light intensity, and photoperiod, remain reasonably unaffected. Here, we present a low-cost, low-input (in terms of construction materials and energy expenditure), field warming setup in which heating cables were combined with a PMMA/acrylic Open Top Chamber (OTC) and show that this setup can effectively raise internal temperatures by ~3 °C-5°C above ambient in field conditions. Assessing shoot phenotypes of cold-tolerant common snowdrops (*Galanthus nivalis*), *Arabidopsis thaliana* natural accessions, and tomato (*Solanum lycopersicum*) confirmed that the OTC setup can be used to study shoot responsiveness to high temperatures in the context of the stochastic outdoor environment. The low-cost materials used, combined with provided construction details and software code, should encourage the swift development of warmed OTCs by researchers worldwide.

## Introduction

Global warming is one of the primary and perhaps most notable effects of climate change, and a further rise in temperature of 0.4-4°C is predicted for the coming decades (Lee et al., 2023). Global warming affects many aspects of plant phenology, development, reproductive traits, biomass accumulation and growth, susceptibility to disease, and more (Chaudhry and Sidhu, 2022; Leisner et al., 2023). Consequently, global climate change has wide-ranging effects on ecosystems and agriculture. Altogether, it is estimated that each °C increase will result in up to 10% yield loss in crops such as wheat, maize, and rice, the main global caloric staple crops (Challinor et al., 2014; Asseng et al., 2015; Zhao et al., 2017; Shahzad et al., 2021; Hultgren et al., 2025). Understanding plant responsiveness to climate change is therefore essential to increasing global plant-based food production and improving food security.

Experiments assessing the effects of temperature increases are therefore particularly important as understanding how temperature changes affect plants is essential for the development of high-producing climate-ready crops that can withstand the negative effects of global warming (Stuble et al., 2021; Rivero et al., 2022).

In order to explore plant responses to increased temperatures, experimental setups that simulate the conditions of a warmer climate are needed. Much work has been done with indoor environments, i.e., in climate-controlled growth chambers and cabinets in labs and in experimental greenhouses (Lippmann et al., 2019; Zhu et al., 2021, 2022; Praat et al., 2024). The precise control of environmental parameters offered by such setups is undoubtedly useful for dissecting the precise effects of temperature on plant responses down to the molecular mechanistic level. However, indoor controlled experiments per definition fail to adequately place temperature effects in the broader environmental context. This is important, since co-occurring stresses often trigger synergistic, additive, or antagonistic effects or form a blend of responses to individual stresses (Morales et al., 2022; Rivero et al., 2022; Jiang et al., 2024, 2025; Xu et al., 2024). Furthermore, the effect of temperature dose (severity) and temporal temperature fluctuations present in natural and agricultural conditions are often neglected (Praat et al., 2021, 2024; Zhu et al., 2022). Connecting the stochastic effects of abiotic and biotic environmental parameters like temperature, precipitation, rainfall, humidity, wind, light intensity, photoperiod, and soil microbiota with temperature responsiveness (Kimball et al., 2008) is imperative if one wants to assess the effects of complex environmental factors and their interaction with temperature on plant growth, development, and phenology. This is the purpose of field warming experiments, artificially raising the temperature of a given area of land (either in the soil, or the air, or both) to mimic natural temperature increases, without interfering significantly with other environmental, weather, and climatic parameters. Since the 1990s, many field warming setups have been designed and tested. These setups have been tailored to various experimental needs and climates, leading to a wide range of designs, efficacies, and energy uses (Ettinger et al., 2019; Frei et al., 2020; Buttler et al., 2023; Hollister et al., 2023). Some have been combined with CO_2_ manipulation and/or precipitation manipulation (**Supplementary Table S1**). In the current field warming literature, areas outside of the USA and Europe are significantly underrepresented (Stuble et al., 2021), leaving gaps in the understanding of plant response to warming in certain climates. Furthermore, many of the most widely used systems (IR heaters and forced air heaters) are high in both material and energy cost, increasing their environmental impact and precluding their use outside of well-funded institutions. Moreover, OTCs are typically placed over existing vegetation (Bokhorst et al., 2008; Sun et al., 2013). While OTCs are used in empirical research, they often unintentionally affect multiple environmental variables.

We aimed for a low-cost low-input (in terms of energy expenditure and material costs) and size-scalable setup. Therefore, IR heaters and most forced-air setups (**Supplementary Table S1**) were excluded. Additionally, the main goal was to alter aboveground air temperature at a height of ~10 cm to assess shoot responses, thus eliminating buried heating cable options, which mainly warm the soil. Considering this, we present the design of an Open Top Chamber (OTC) field warming setup with suspended heating cables, capable of generating three to five degrees of warming above the prevailing ambient temperature in the temperate climate of The Netherlands from winter to summer. We show that our setup triggers expected changes to thermoresponsive traits, such as increased biomass accumulation, elongation of stems, hypocotyls, and flowering, in common Snowdrops, *Arabidopsis thaliana*, and tomato plants. We present design, construction, and operational details allowing colleagues to build their own OTC setups. The low construction costs will be helpful to scale up the OTC and build several setups, allowing complex multifactorial experiments in experimental fields and in natural vegetation. This undoubtedly will result in new insights into effects of high temperature on plants under otherwise natural and stochastic climatic and weather conditions.

## Materials and methods

### OTC physical construction and specifications

A list of materials used for OTC construction with relevant information can be found in **Supplementary Table S2**. Each OTC is composed of six trapezoidal PMMA panels with the following dimensions: 5-mm thickness, 49-cm height, 108-cm bottom width, and 80-cm top width. These panels are connected to form a hexagonal OTC. The angle of the panels relative to the ground is 60°. The panels were connected by strips of stainless steel bolted to the panels. The specifications of the steel connectors are as follows: 5-mm thickness, 50-cm height, 8-cm width. The steel strips have two 5-cm triangular teeth cut into their bases to secure the OTC to the ground and are bent vertically along the midline to an angle of 120°.

The heating cables used are 18-W/m resistance wire cables intended for underfloor heating (7423418520541, Decochip, The Netherlands). The cables used are cut to lengths of 4m with a total maximum power output of 72 W per cable. In the final field setup (Layout #4, placed outside), there are a total of eight 4-m cables with a total length of 36m and a total wattage of 576 W.

### OTC electronics layout

The entire system runs on 230V and is connected to a regular (outdoor) power socket. The electronics for this system (**Supplementary Figure S1**) consist of two parts. The first part is the heating cable power supply and control. The second part is the thermocouple data logger (used for indoor experiments). Circuit diagrams and overall electronics layout can be found in **Supplementary Figures S1** and **S2**. All components of the setup are controlled by means of an Adafruit Feather HUZZAH ESP8266 microcontroller (Adafruit Industries, New York, NY, USA). The heating cables are powered by two 24-V DC power supplies (Mean Well Inc, Fremont, CA, USA). Each power supply powers four heating cables. The heating cables are controlled by the microcontroller using IRLZ34NPbF MOSFETs (Infineon Technologies AG, Neubiberg, Germany). These electronic switches can be used to switch the two groups of cables ON/OFF and can function potentially as dimmers allowing for future feedback regulation in the system. ESP8266 can be controlled locally by connecting to the local Wi-Fi network it broadcasts and navigating to its webserver. The webserver provides basic status information and enables ON/OFF switching of either group of heating cables. The MOSFETs are cooled by two 230-V fans (Sinwan, Electric Industries Co, Taipei, Taiwan). The thermocouple data logger consists of an SD card logger with a real-time clock (Adalogger Featherwing, Adafruit Industries, New York, NY, USA), thermocouple amplifier boards (Adafruit MCP9600 I2C Thermocouple Amplifier, Adafruit Industries, New York, NY, USA), and type T thermocouples (Labfacility, Dinnington, UK). The thermocouple data logger runs using a set of custom-built Arduino code that can be found here (https://github.com/catoprovector/Field-Warming-Project).

For the indoor experiments, the electronics were connected using breadboards and snap connectors (WAGO Group, Minden, Germany). In preparation for moving the setup outside, the electronics layouts were finalized and moved from prototyping breadboards and snap connectors to soldered circuit boards and more robust connectors. Fuses were added to every individual heating cable, as well as an IP44-rated GFCI. Waterproof insulation was added to the heating cables including custom-printed cable connectors (3D printing information can be found here: https://www.thingiverse.com/thing:6241330).

To house the electronics that could not be directly waterproofed, a white wooden hutch was built (**Supplementary Figure S3**). This hutch was made of 18-mm plywood and has base dimensions of 60 × 60 cm and a sloped roof of 68 × 68cm, the overhang of which prevents water from seeping into the inside of the hutch. The hutch was raised 10cm off the ground on 45-mm dowel legs. Ventilation holes in the bottom of the hutch along with a 24-V computer fan located just under the top of the overhang served to provide airflow within the hutch to prevent any electronic components from overheating. Fine screen mesh was fitted over the vents to prevent ingress by insects or other animals. Two ports for wires were cut into the base of the hutch and fitted with flanged rubber discs on both the inside and outside of the base wood to allow cable access while restricting animal ingress.

### Indoor OTC validation

**Figure 1:**
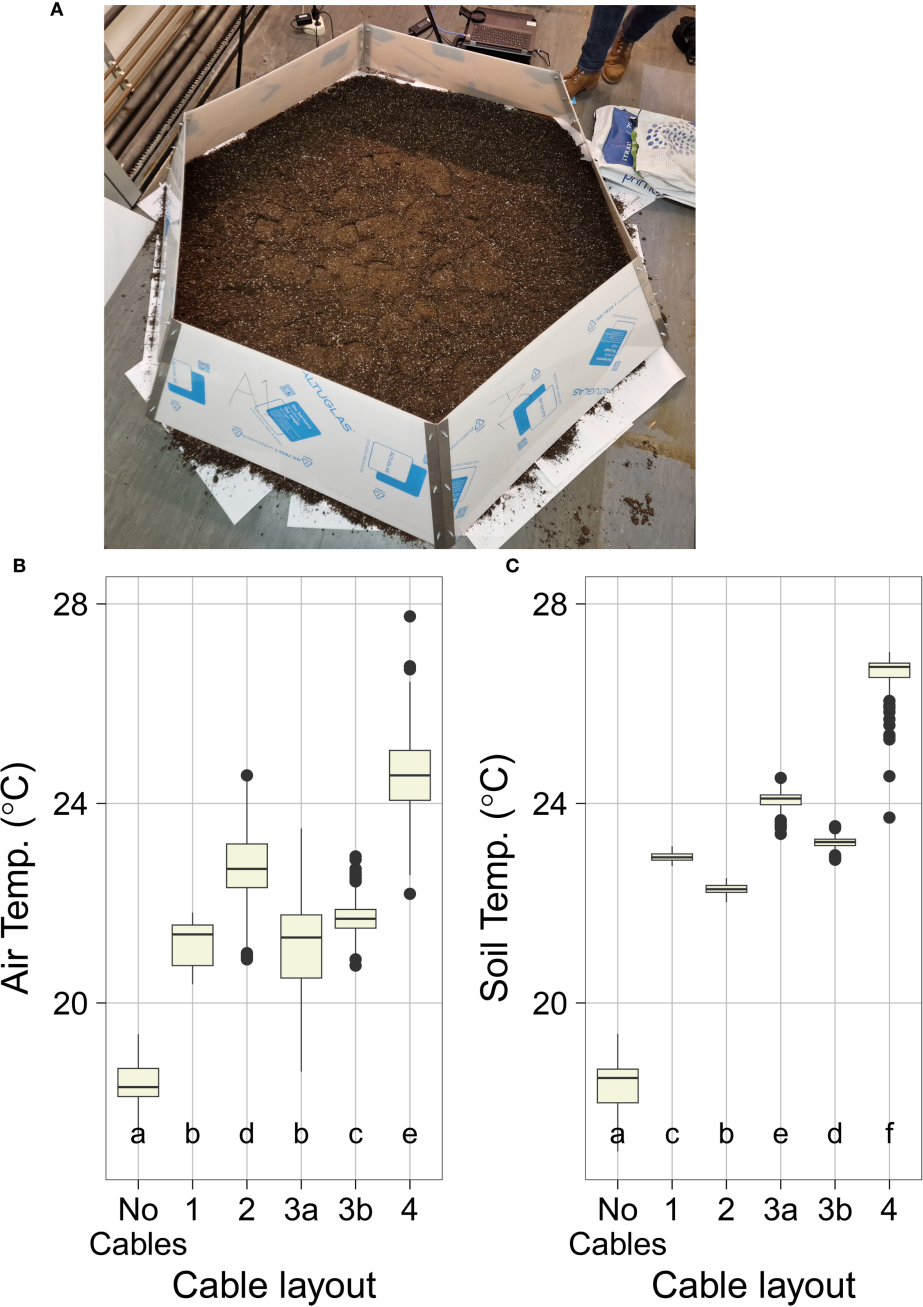
OTC construction and indoor validation. **(A)** Overview of constructed OTC setup placed indoors for validation purposes. Note that the PMMA walls are here covered with white foil to protect the setup from scratching during validation, construction, and transport. Also note the layer of potting soil that was placed inside the setup to aid soil temperature quantification. **(B, C)** Performance of different OTC heating cable setups (#1, #2, #3A, #3B, and #4) and no cables control during indoor validation (see also **Supplementary Figures S4**-**S6**). Presented values are a subset of the total experimental data using only the data points from the heated portion of each validation experiment. Indicated are **(B)** internal OTC air temperature, measured with thermocouples at random spots inside the OTC (control thermocouples placed outside the OTC are not considered here). Data are averaged read-out of three to four thermocouples obtained during the test runs. **(C)** Soil temperatures as obtained by IR thermography image analysis of five measured spots perpendicular to each other (up, down, left, right, and center area of the plot, avoiding parts with heating cables crossing). Boxes indicate boundaries of the second and third quartiles (Q) of the data distribution. Black horizontal bars indicate median and whiskers Q1 and Q4 values within 1.5 times the interquartile range. Numbers below the bars indicate *p*-values (unpaired t-test), with letters indicating different significantly different groups (*p*<0.05, TukeysHSD).

**Figure 2:**
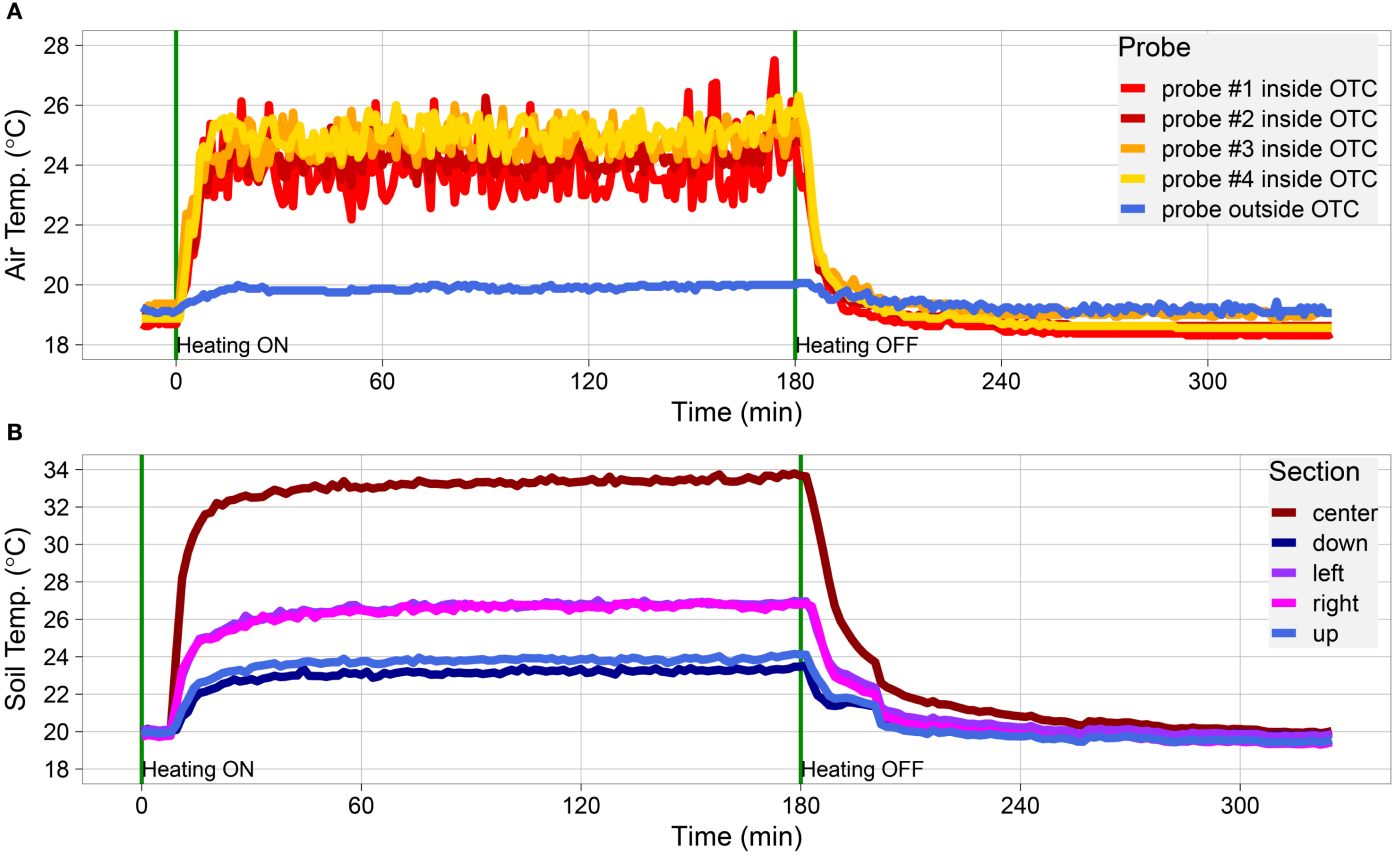
Dynamic performance of cable layout #4 during indoor validation. **(A, B)** Temperature data measured inside the OTC placed indoors. **(A)** Data obtained with thermocouples placed at random spots inside the OTC (thermocouple probes #1-4; indicated in red-to-yellow) and a thermocouple placed outside of the OTC (thermocouple, indicated in blue; representing the (baseline) test room temperature). **(B)** Soil temperatures as obtained by IR thermography. Spot measurements were taken from the overall thermal picture capturing the whole OTC, in the center of the OTC, and four spots perpendicular to each other in between the center (brown) and the edge of the OTC, indicated as down (dark blue), left (purple), right (pink), and up (light blue). Spots with heating cables crossing were avoided in the measurements. Cables were switched on at t = 0 min and switched off at t = 180 min, indicated by a vertical green line.

Indoor tests were performed using a single OTC placed indoors in our laboratory building in a well-ventilated climate stable room in the core of the building, deprived of windows (**Figure 1**, **Supplementary Figure S4**). The OTC was placed on a layer of approximately ± 1 to 2cm of Primasta potting soil. Over the course of several subsequent experiments, different cable setups were assessed for adequate heating, with layout #1–3 containing four cables and a total wattage of 288 W and the final layout #4 containing eight cables having a total wattage of 576 W. The following setups were assessed (**Supplementary Table S3**, **Figure 2**, **Supplementary Figures S4**-**S6**):

- *No heating*: mimicking the OTC^w−^ setup lacking heating cables. Temperature data were collected over the course of 3 days.
- *Layout #1*: Similar to the layout in Sun et al. (2013). 12m of heating cable was wrapped around a hexagonal PVC support with a perimeter of 5.54m. This PVC support was suspended approximately 10cm above the ground within the OTC, parallel, and in proximity of the PMMA walls.
- *Layout #2*: Random distributed network of heating cables suspended ~8–12 cm above the ground on a PVC and wire frame. This cable setup resembled a spider web.
- *Layout #3a+b*: Distributed spoke and wheel layout with the cables lying on the ground (layout #3a) or suspended from the PVC and wire frame ~8–12 cm above the ground (layout #3b). In both layouts, one cable went around the perimeter of the OTC and the other cables were laid from vertex to vertex of the hexagon dividing it into six smaller triangles.
- *Layout #4*: Combination of layout #3a plus #3b wiring (on the ground and suspended).

### Temperature data recording and analyses

Air temperature data for the five tested cable layouts were collected using thermocouples with an accuracy of ±0.5˚C (Labfacility, Dinnington, UK). For each experiment, three (no heating, layouts #1, #2) or two (layouts #3a, #3b, #4) thermocouples were placed randomly within the OTC. Ground temperature data were recorded with an IR thermal camera (FLIR A600 Series) that was mounted above the OTC (**Figure 2**, **Supplementary Figures S5**, **S6**). For the OTC^w+^ experiments, the thermal camera and thermocouples were activated well before the cables were turned on to capture the ramp-up period. Once the cables had run for 1-3 h, they were switched off and the system was monitored for at least an hour longer to capture heat dissipation.

IR thermal data were captured every 94.66 s, and data produced by the camera were exported as a.CSV file where each pixel (480 × 640) represents a single temperature reading. Images were analyzed using R by taking five 75 × 75 pixel snapshots across each image. These snapshots were laid out with one snapshot in the middle, and one to the left, right, top, and bottom closer to the edge of the OTC as determined by visual inspection of the images. This R script then cycled through all the.CSV files for a given experimental run pulling out the min, max, and mean temperatures from each of these sections as well as removing any data points above an arbitrary value of 40°C, as temperatures above these were roughly double the expected temperature and could only be produced by the cables themselves. These datapoints were therefore not included in the analysis. This process produced a timeseries of data points for each experimental run.

### OTC field plot establishment

**Figure 3:**
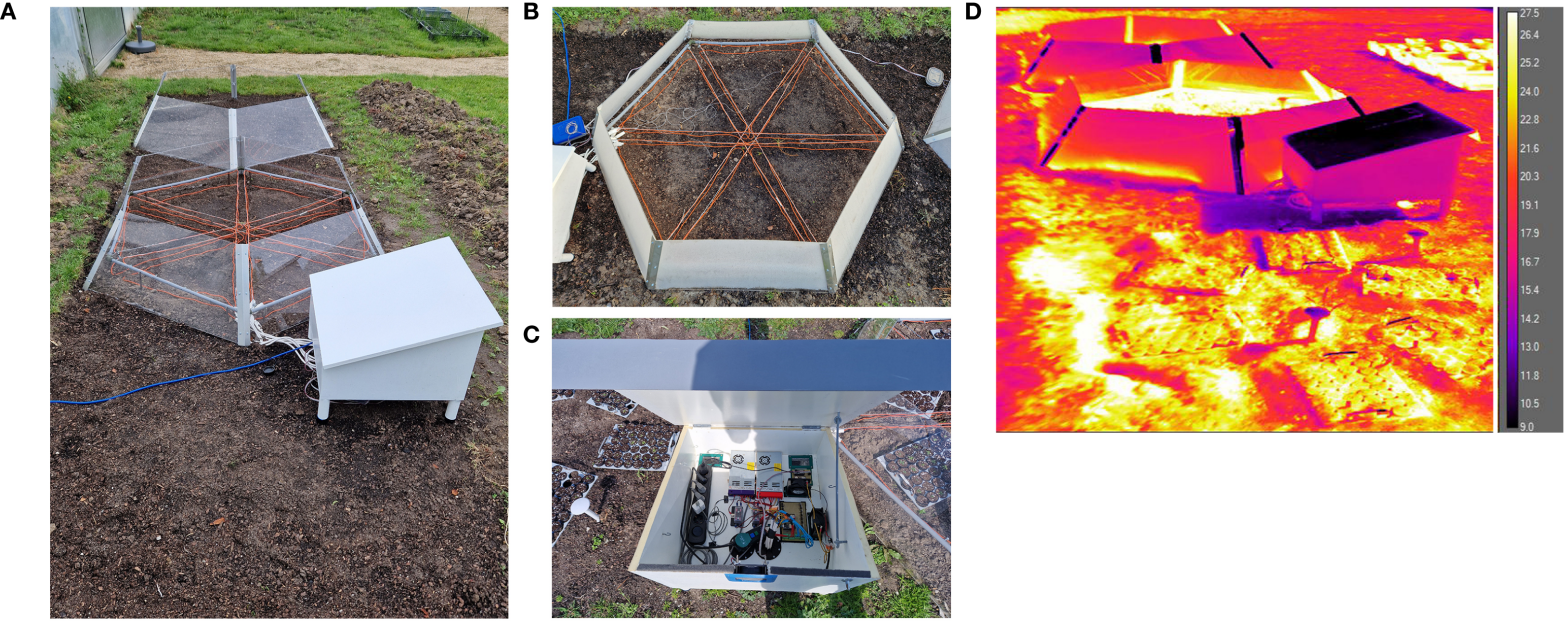
OTC setups placed in the field. **(A)** Overview of field site with the empty control plot **(C)** in the front, the OTC^w+^ in the middle, and OTC^w−^ in the back. **(B)** Top view of OTC^w+^ illustrating cable layout #4 (orange wires are the heating cables). **(C)** Electronics layout in the plywood hutch; see **Supplementary Figure S1** for details). **(D)** IR thermography image of the control plot and OTC setups, illustrating the effectiveness of the OTC^w+^ (middle). Scale bar indicates the detected temperatures (°C).

The OTCs and empty control plot were established in the Botanic Gardens at Utrecht University at the coordinates 52°0″23.″″N 5°1″21.″″E (**Figure 3**, **Supplementary Figure S3**). Weeds were removed *a priori*, and the existing soil (mix of dense and loose clay) was turned over and mixed with some loose highly organic soil to form a plot of 2 × 6m. Within this larger plot, the two OTCs and the control plot were assembled with the heated OTC^w+^ in the middle and the longitudinal axis across the three plots facing ~south–south-west. Per setup/plot, two TOMST^®^ DS7505U+ microclimate loggers (Wild et al., 2019) were installed that continuously measure temperature 10.3cm belowground, at the soil surface (soil/air interface) and 12.5cm aboveground, plus soil moisture (10.3cm belowground). The TOMST microclimate loggers were read-out using a TMD adaptor and a laptop. Effectiveness of the warming was verified using an IR thermal camera (FLIR A600 Series) (**Figure 3D**, **Supplementary Figure S7**).

### Plant materials and trait assessment

#### Snowdrop (*Galanthus nivalis*)

Snowdrops were used in our experiments as representation of cold-tolerant species (development occurs in late winter, within a thermal range of −0.8 °C to +8.5 °C), enabling to test the performance of our OTC setup in Dutch winter conditions. Common snowdrop bulbs (*Galanthus nivalis*) were collected with permission on August 14, 2023, from the ‘Niënhof’ estate (Bunnik, The Netherlands) located close to the UU Botanical gardens and directly planted in a random manner distributed over the OTCs and control plot at a depth of ~5 cm. The moment the shoots emerged from the soil (January 22, 2024), the heating cables of the OTC^w+^ were switched on for the remainder of the experimental period. On February 13, 2024, shoots were collected and photographed for visual comparison. No watering was applied during the entire experimental period.

#### Arabidopsis thaliana

*Arabidopsis thaliana* was used in our study as the species has an extraordinary capacity and predictable response to changing temperatures and is *the* accepted laboratory model for studies on plant genomics and molecular genetics (Zhu et al., 2022; Quint et al., 2016). A total of 21 Arabidopsis accessions were used for hypocotyl length and phenology assays. There were 11 accession derived from the Iberian peninsula collection (Cap-1, Bea-0, Bis-0, Don-0, Fei-0, Gra-0, Ale-4, Rab-7, Bus-0, Pig-0) and obtained from several Spanish regions with elevation differences. These accessions represent wide diversity in temperature patterns in our OTC experiments (Picó et al., 2008; Castilla et al., 2020; Subrahmaniam et al., 2025). There were 10 accessions collected locally in the vicinity of Utrecht (**Supplementary Table S4**), The Netherlands, relatively close to the location of our OTC setups. These accession enables testing effects of elevated temperature on locally-evolved plants. To obtain fresh and homogenous seed batches, plants were first re-propagated in an indoor LED growth chamber set at 21°C, 110-130 µmol m^−2^ s^−1^ photosynthetically active radiation (PAR) at a 16-h photoperiod (8 h darkness). When the plants were 3 weeks old, they were vernalized at 4°C at dim light for a month and thereafter placed back in growth chamber conditions and allowed to complete the life cycle and seeds were harvested.

For hypocotyl length quantification, re-propagated seeds were sterilized using a solution of 0.8% commercial bleach (Glorix) in ethanol for 10min, followed by twice washing with ethanol for 10min and thereafter taken up in water and stratified (4 °C in the dark) for 4 days. Subsequently, 20 seeds divided over two plates (containing 10 seeds each) of each accession per treatment (OTC^w+^ and OTC^w−+^, cabinet 10°C and cabinet 20°C) were placed in rows on Petri dishes containing 1% plant agar medium with full-strength Murashige–Skoog (MS including MES Buffer and vitamins, Duchefa) without sucrose. After containing the plates o/n at 4 °C, they were placed either outdoors on March 26, 2024, in the OTCs and control plot (**Supplementary Figure S8**) or in climate-controlled cabinets in the lab, set at 10 °C or 20 °C under a 10-h photoperiod, 110-130 µmol m^−2^ s^−1^ photosynthetically active radiation (PAR) and RH of 70%. On April 2, 2024, the (8-day-old plant) plates were scanned using a regular PC-connected flatbed scanner. Hypocotyl length was measured using ImageJ.

For phenology (bolting and flowering time) assessment, Arabidopsis seeds were sown on 9 × 9cm pots and stratified for 3 days at 4°C and thereafter placed under a transparent lid in the indoor LED growth chambers with settings as described above, except that the temperature was 15°C. After 9 days, the germinated seedlings were transferred to round (5 × 5cm) pots containing Primasta potting soil. For each plot, six groups of plants were prepared (42 plants per group) in custom-made carrying trays (with no bottom), with two replicates of each accession per group, placed at a random position within the group (in total 12 plants per accession per environmental condition). Plants were kept another 6 days in the indoor LED growth chambers with settings as described above at 15°C and were then transferred to the outdoors empty control plot and OTCs (**Supplementary Figure S8**) on March 4, 2024. A total of 14 plants (of a total of 756 plants) were omitted due to poor seedling establishment after transplanting.

From the moment the first plant started bolting (April 10, 2024), bolting time (day bolt visible) and flowering time (day the first flower opened) were scored three times a week on regular intervals until the moment that the last plant flowered (May 20, 2024). No watering nor other interferences were applied during the experimental period. In total 395 plants survived until flowering, with significantly more local accessions surviving than Iberian peninsula accessions (T.Test; *p* =0.0061).

#### Tomato (*Solanum lycopersicum*)

Tomato was used in our study to showcase the effectiveness of our OTC setup to study the effects of high temperature on crops. In addition, since optimal tomato growth and performance occurs at relatively warm conditions (above 20°C) (Adams et al., 2001), the use of tomato enabled testing our OTC setup in Dutch summer conditions. Tomato seeds (cultivar Moneymaker, LOT.C.20171-3) were commercially obtained from www.moestuinland.nl, brand “Sluis Garden”, and planted in 9 × 9cm pots containing wetted Primasta soil at a depth of 1cm and placed in an indoor LED growth chamber set at 21°C, 110-130 µmol m^−2^ s^−1^ photosynthetically active radiation (PAR) at a 16-h photoperiod (8 h darkness) under a transparent lid. After 5 days, ~25% of the seeds germinated and three equal groups were made based on fraction of germinated seeds and seedling size on May 8, 2024. Directly after assigning to the experimental groups, the plants were moved outside to the OTCs and placed in either the empty control plot (in total 52 seeds/seedlings), the OTC^W+^ (in total 51 seeds/seedlings), or OTC^w−^ (in total 56 seeds/seedlings). Plants were left to germinate and develop until the moment the first leaf emerged. At that moment the seedling was gently removed from the pot, the hypocotyl was measured with a caliper and the seedling was placed in the bare soil at a random position in the respective control plot or OTC (**Supplementary Figure S9**). This soil was locally wetted prior to seedling transfer to benefit establishment. Additionally, the plants were watered twice a week, and this frequency was temporarily increased during the second week of May (2024) because due to a heatwave the soil dried out quickly. At the moment that the first plants developed visual floral buds, the shoots of the plants were cut at the shoot/root base (June 27 and 28, 2024). Leaves were removed, and the number of internodes per plant were counted and the internode length was measured with a digital caliper.

## Results

### Field warming setup: design principles and considerations

The goal of this project was to create a functioning semi-controlled field warming setup that can effectively and consistently warm the air surrounding plant shoots (aboveground). After a literature survey (**Supplementary Table S1**), it was decided to use a combination of hexagonal OTCs fitted with 18-W/m resistance wire heating cables powered (24V DC) from a separate water-proofed box and operated by an ESP8266 microcontroller (**Figure 3**). The reasoning was that basic (passive, unheated) OTC designs are relatively easy to execute but are ineffective and inconsistent at warming except when in direct sunlight (Johnson et al., 2013; Sun et al., 2013). Additional design considerations were as follows: 1) the setup should be built within a budget of €1500 (year 2022) and 2) the setup should be scalable and extendable, both in physical size and in technological capability (e.g., the possibility of adding more heating, adding (de)moisturizers, more sensors, applying feedback control, and applying remote monitoring and control).

The inspiration for the OTC design came from Bokhorst et al. (2008) and Sun et al. (2013) but needed modification to fit our requirements. The Sun et al. (2013) design used UV transparent PMMA, whereas our setup uses regular, partially UV-blocking, PMMA. In the experiments conducted by Bokhorst et al. (2008) and Sun et al. (2013), the OTCs were placed over existing vegetation, meaning that every square cm of OTC area was relevant for their study. Our current setup is primarily meant for experimental research in which the plants are added after the OTC setup has been installed. This allows for the plants to be placed in specific locations, for instance by leaving the areas under the angled walls unplanted to avoid edge effects. The additional controls of an unheated OTC (passive warming) and completely bare plot (empty control) would provide data to help correct for effects of the PMMA shadow. Because of this, the UV shadow area of the OTC PMMA sides was not considered a major issue as it could be circumvented and/or corrected for. We thus decided to construct two identical OTCs: a warmed one supplied with heating cables (referred to as OTC^w+^) and a non-warmed one (referred to as OTC^w−^), supplemented with an empty control plot (C) in the field, which allows to estimate/account for passive warming effects that may occur in both OTCs.

### Construction and indoor validation of the OTC setups

Compared with the setups described in Bokhorst et al. (2008) and Sun et al. (2013), the PMMA thickness used in our OTCs was increased from 4 to 5mm for two reasons. First, the additional 1mm decreased IR transmissivity of the PMMA without dramatically decreasing the visible-light transmissivity, thereby increasing the thermal insulation of the OTC. Second, the increased thickness and rigidity allowed for a simplified OTC design by eliminating the top and bottom metal supports used in the Bokhorst et al. (2008) setup, without significantly compromising structural integrity.

After construction, tests were performed using a single OTC to test the efficacy of different OTC-heating cable layouts under otherwise stable environmental conditions. For this purpose, the OTC was placed indoors in our laboratory on a layer of ±1 to 2cm of potting soil (**Figure 1A**, **Supplementary Figure S4**). In total five different OTC^w+^ cable layouts were tested, beginning with the layout used in Sun et al. (2013). For each experimental run, temperature data were collected using three to four thermocouples placed in several positions in and outside the setup (**Figure 2A**, **Supplementary Tables S3**, **S5**). In parallel, ground temperature was recorded by spot measurements using a FLIR IR-thermal camera (**Figure 2B**, **Supplementary Figure S5**, **Supplementary Table S6**). An initial 3-day run without applied heating (effectively the OTC^w−^ setup) confirmed that air and ground temperatures inside the OTC were very stable during this experiment (**Supplementary Figures S5A**, **S6**), with some minor variations in temperature that can be attributed to temperature fluctuations in the laboratory building (mainly differences between day and night).

Cable layout #1 consisted of a hexagonal PVC frame wrapped with heating cables along the bottom inside perimeter of the OTC (**Supplementary Figures S4A**, **S5B**). This layout replicated the one described in Sun et al. (2013). Cable layout #2 consisted of a random arrangement of the cables over a suspended PVC and wire frame, creating a spiderweb like structure (**Supplementary Figures S4B**, **S5C**). A major drawback of this layout #2 is that it limits access to the planting surface of the OTC. Cable layout #3a consisted of an orderly positioning of cables directly on the ground in a spoke wheel pattern. This layout provided sufficient areas of exposed ground in which to plant always in relatively close proximity to the heating cables (**Supplementary Figures S4C**, **S5D**). Cable layout #3b was the exact same as cable layout #3a, except that the cables were suspended ~10 cm above the ground (**Supplementary Figures S4D**, **S5E**). Cable layout #4 was a combination of the layouts #3a and #3b with cables both on the ground and suspended in a spoke wheel pattern (**Supplementary Figure S5F**). Notably, combining both cable positions doubled the number of cables in layout #4 bringing the total power to 576 W as compared with the 288 W in layouts #1-3.

All OTC^w+^ layouts led to increased air temperature inside the OTC, with values ranging from 2.83°C (layout #1) to 6.15˚C (layout #4) above ambient, compared with the OTC^w−^ no-cables control (**Supplementary Table S5**). A similar trend was seen in the soil temperature data (**Supplementary Figure S5**, **Supplementary Table S6**). Compared with layout #1, layout #2 was marginally more effective at heating the air (1.52°C increase) and slightly less effective at heating the soil (0.64°C lower on average) (**Figures 1B, C**, **Supplementary Tables S5**, **S6**). The proximity of the thermocouples to the cables likely played a role in the temperature differences observed across the remaining layouts. In layout #2, the suspended cables were closer in proximity to the thermocouples (raised approximately ~10 cm from the ground) than in layout #3 where the cables were at ground level. As a result, recorded air temperatures layout #2 were higher, but soil temperatures were lower as compared with layout #3 (**Figures 1B, C**, **Supplementary Tables S5**, **S6**). Layout #3b performed comparably with layout #3a in both air temperature and soil temperature, indicating effective air circulation within the OTC.

Layout #4 was superior to the other layouts in increasing both air and ground temperatures (**Figures 1B, C**, **Supplementary Tables S5**, **S6**). This is likely due to the doubling of the heating cables as compared with the other setups. The compiled data show that, more than any specific cable layout, the primary driver of overall OTC^w+^ temperature is raw energy input. Consequently, layout #4 was used for further investigation of secondary system parameters such as heating speed, heat retention, and heat dissipation (how quickly the system heats up and cools down), and thermal distribution within the OTC^w+^. Both the rapid heating speed and the low heat retention were immediately obvious (**Figure 2**). The cables reached near-peak heating within ~10-15 min of being turned on, and internal OTC^w+^ temperatures adjusted with the same rapidity (**Figure 2**). The warmth, likewise, dissipates swiftly from the OTC once the cables are shut off (5-30 min). This indicates that the system requires continuous energy input to maintain constant elevated temperatures. The speed of dissipation also suggests a highly dynamic system, which would allow for very fine control of temperature if coupled with feedback regulation.

### Outdoor validation of the OTC setup

**Figure 4:**
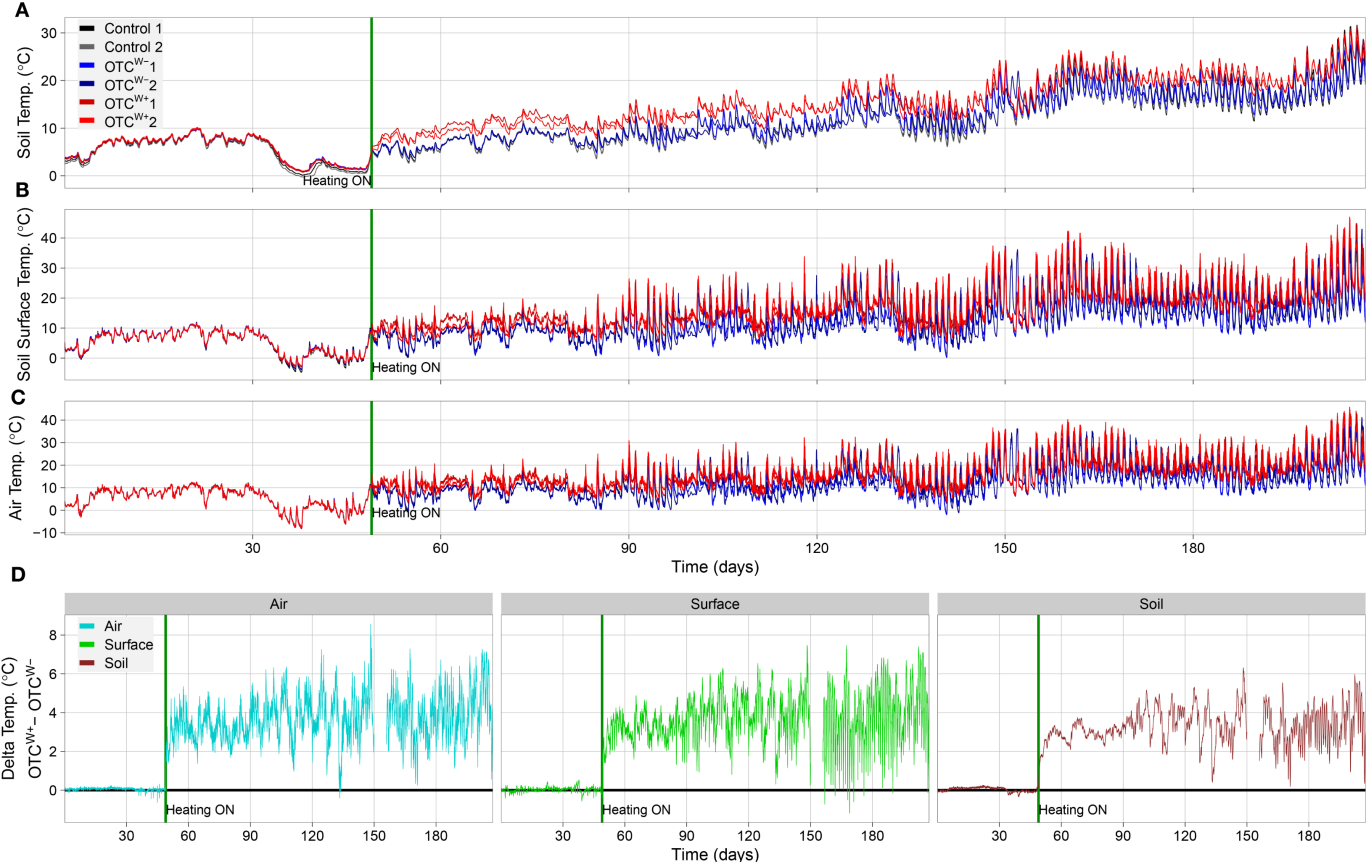
TOMST microclimate logger readouts during the project period. **(A-C)** Temperatures (°C), **(A)** 10cm below ground (soil temperature), **(B)** at the soil surface (soil/air interface), and **(C)** 10cm above the ground during the experimental period (air temperature). The TOMST microclimate sensors were placed in the plots on December 14, 2023 (day 0), and the OTC^w+^ was switched on, on January 22, 2024, indicated by a green vertical line in each panel representing. Note the immediate rise in temperatures in the OTC^w+^ after switching on (red lines, each line representing an individual TOMST sensor) compared with the OTC^w−^ (blue lines) and empty control plot (gray lines; visually masked by near-identical values in the OTC^w−^ for most of the experimental period. Also note the differences between day and night temperatures, and how this is relatively well buffered in the soil (10cm below ground) compared with air and surface temperatures *and* the gradual increase in temperature over the seasons from winter (left part of the panels) to summer (right part of the panels). **(D)** Temperature difference between OTC^w+^ and OTC^w−^ throughout the experimental period above ground (left panel; blue line), at the soil/air interface (green line; middle panel) and below ground (right panel; dark red line). missing data between day 150 and 156 represent a systems failure wherein data were not correctly logged.

Following the indoor validation, the OTCs were assembled outside in the Utrecht University Botanic gardens after plots were cleared of weeds. The OTC^w+^ plot was equipped with cable layout #4 (**Figure 3**, **Supplementary Figure S3**). Each plot was monitored by two TOMST microclimate loggers (Wild et al., 2019) (**Supplementary Figure S3C**) that continuously measured temperature belowground (soil temperature), at the soil surface (soil/air interface) and aboveground (**Figure 4**). The TOMST microclimate loggers were installed on December 14, 2023, and activated on January 22, 2024 (**Figure 4**). Clearly, the temperatures were highly similar between all plots and sensors in the time period before switch-on. Since December-January is midst winter in The Netherlands, no passive warming effect was noted in the OTCs compared with the empty control (C) plot. After switching on the OTC^w+^, the temperature immediately increased compared with both the OTC^w−^ and C plot, which persisted throughout the experimental period (**Figure 4**). This warming effect was most notable in soil temperature (**Figure 4A, D**). This is likely because soil is better buffered against temperature changes than air, being more prone to diurnal temperature changes (day/night effects) and stochastic weather influences (*e.g.*, temperature dissipation by wind) (**Figure 4**). IR thermography confirmed the contribution of both passive and active warming to increased temperature inside the OTCs (**Figure 3D**, **Supplementary Figure S7**).

### Effect of field warming by the OTCs on plant development and phenology

As a proof of principle, we next tested if our OTC setups are an effective tool to study the effects of global warming on plant growth, development, and phenology. To this aim, the responses of cold-tolerant snowdrops (*Galanthus nivalis*), the plant laboratory model *Arabidopsis thaliana*, and the commercial crop tomato (*Solanum lycopersicum*) to OTC warming were assessed in their appropriate growing seasons: Dutch winter, early and late spring and (early) summer, respectively.

### Snowdrops (*Galanthus nivalis*)

Common snowdrop (*Galanthus nivalis*) is a typical cold-tolerant species whose growth and development occurs in late winter, within a thermal range of −0.8 °C to +8.5 °C. Development and morphology in *Galanthus* are highly temperature dependent (Sparks et al., 2006). For instance, stem and leaf elongation tightly scale with temperature input (Abrami, 1972) and shoot length is inhibited by ~50% in plants grown at −1°C when compared with those grown at 6°C (Orthen and Wehrmeyer, 2004).

**Figure 5:**
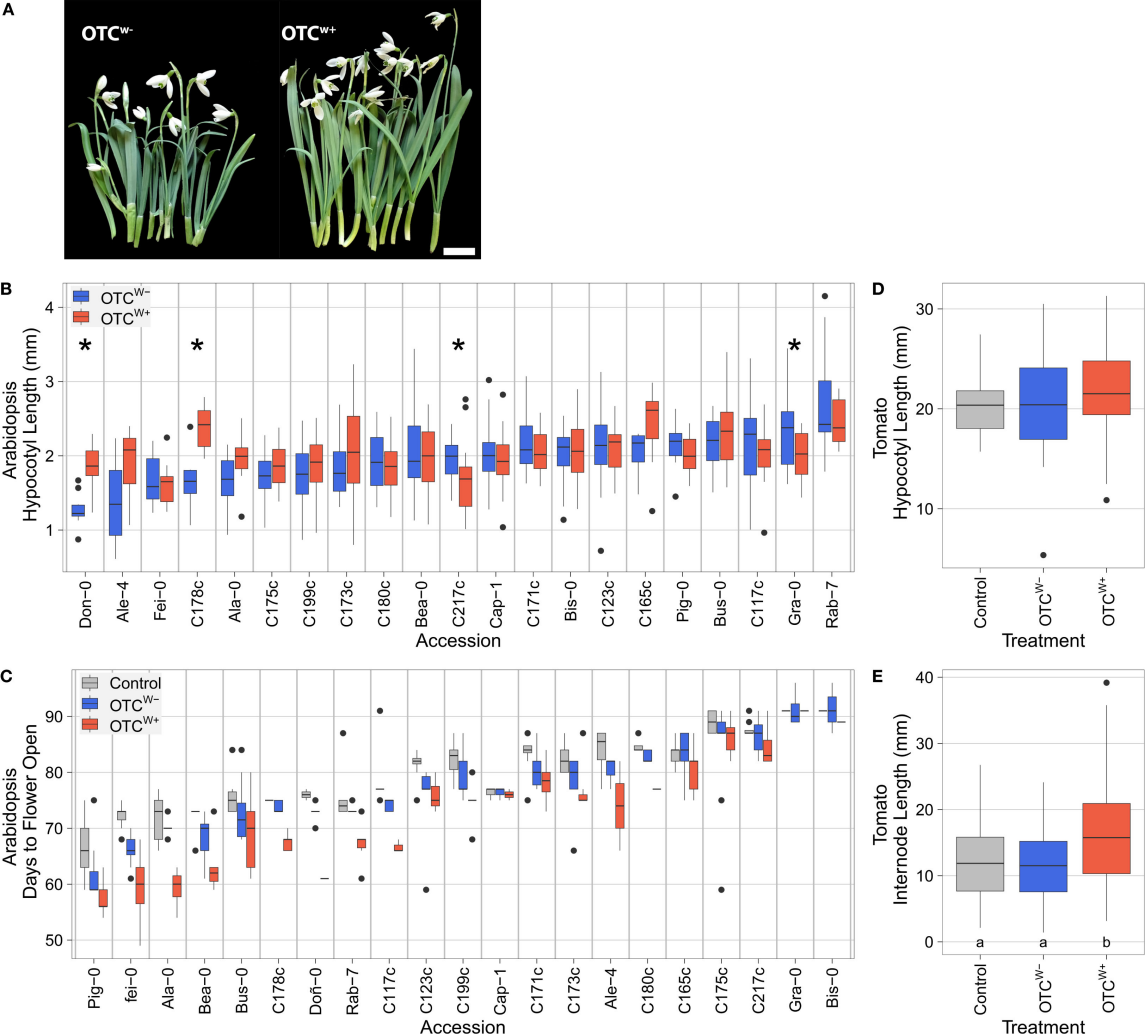
Effect of OTC field warming on plant development and phenology. **(A)** Field warming stimulates development of *Galanthus nivalis. Galanthus nivalis* accession Niënhof bulbs were planted in the OTC^w+^ and OTC^W−^ setups on August 14, 2023. At the moment the snowdrops emerged (January 22, 2024), the warming cables were switched on. Plants from the warm OTC^w+^ (right) and ambient temperature OTC^w−^ were harvested 22 days later and photographed on February 13, 2024. Note the clear elongation of leaves and floral stems triggered by the mild warming imposed by the OTC^w+^. Scale bar = 1cm. **(B)** Hypocotyl lengths of 8-day-old seedlings (n = 4-24; median n = 18, significant differences at *p*<0.05 (unpaired t-test) are indicated with an asterisk. When no asterisk is shown, the difference was not significant) and **(C)** numbers of days until the moment the first flower opened (n = 1-11; median n = 7; ANOVA: genotype p < 1e−16, treatment: p < 1e−16, interaction p = 0.07), of Iberian peninsula and locally collected *Arabidopsis thaliana* accessions. **(D)** Tomato hypocotyl length (n = 34-48; no significant differences between groups; C vs. OTC^w−^ *p=*0.98; C vs. OTC^w+^ *p=*0.106; OTC^w−^ vs. OTC^w+^ *p=*0.184, TukeysHSD). **(E)** Internode length (n = 148-212). Numbers below the bars indicate *p*-values (TukeyHSD), with letters indicating statistical significantly different groups. **(B-E)** Plants were placed in the OTC^w−^ (blue) and OTC^w+^ setup (red) or the empty control plot (gray). Boxes indicate boundaries of the second and third quartiles (Q) of the data distribution. Black horizontal bars indicate median and whiskers Q1 and Q4 values within 1.5 times the interquartile range. Dots represent outliers beyond Q1 and Q4.

We collected dormant snowdrop bulbs from the “Niënhof” estate (Bunnik, The Netherlands) and planted them in the OTCs in late summer of 2023. As soon as the shoots emerged from the soil, the warming cables of the OTC^w+^ setup were activated (January 22, 2024; **Figure 4**). In the following 3-week interval, the average aboveground temperature inside the OTC^w+^ was 11.18 ± 0.53°C, exceeding that of the OTC^w−^ by 3.62°C (**Supplementary Figure S10A**). Similar effects were noted on soil and soil/air interface temperatures. The temperature differences between the OTC^w−^ and the control (C) plot was negligible (aboveground difference; 0.01°C) (**Supplementary Figure S10A**), probably due to a lack of sunlight during the winter prohibiting passive warming. We observed that the plants in the OTC^w+^ exhibited considerably longer leaves and floral stems than those in the OTC^w−^ plot (**Figure 5A**), showcasing the effectiveness of active OTC warming to study plant development of cold-tolerant species in a realistic global warming scenario.

#### *Arabidopsis thaliana* seedling responsiveness; hypocotyl length assays

Next, we assessed whether our field warming setup could be used to study temperature responsiveness of *Arabidopsis thaliana* under natural conditions. Arabidopsis typically flowers in spring in The Netherlands, with peak intensity late April. We first quantified seedling hypocotyl length, a hallmark trait of thermomorphogenesis (Quint et al., 2016; Ibañez et al., 2017; van der Woude et al., 2021; Delker et al., 2022), which is known to scale with temperature input (Ibañez et al., 2017; Zhu et al., 2022; Praat et al., 2024). In addition, hypocotyl elongation capacity is a reliable predictor of plant trait responsiveness to temperature later in the plant’s life, including flowering time that is typically accelerated at warm temperatures (Ibañez et al., 2017). Seedlings of 21 selected accessions (**Supplementary Table S4**) were placed in the OTCs between the end of March 2024 and the beginning of April 2024, on MS-agar-containing Petri dishes (**Supplementary Figure S8**). The same experiment was conducted in parallel using indoor climate cabinets set at 10°C and 20°C. On average, the aboveground temperature of the OTC^w+^ was 14.93 ± 0.72°C and exceeded the OTC^w−^ temperature by 4.87°C during the experimental period (**Supplementary Figure S10A**). Similar differences were noted for soil and soil/air interface temperature, whereas the temperature difference between the OTC^w−^ and the control (C) plot was negligible (aboveground difference; 0.18°C) (**Supplementary Figure S10A**). The indoor climate cabinet experiment indicated that 14 out of the 21 accessions were sensitive to warming (at dT=10°C), as indicated by significant hypocotyl elongation (**Supplementary Figure S11A**). In the outdoor OTC^w+^, four accessions had a significantly longer hypocotyl (at dT=4.87°C) (**Figure 5B**). Of note, hypocotyls remained overall shorter in the OTCs compared with the indoor experiments (compare **Supplementary Figure S5B** with **Supplementary Figure S11A**).

#### *Arabidopsis thaliana* phenological response; survival, bolting, and flowering time assays

To quantify the effects of field warming on phenology, plants of the same 21 Arabidopsis accessions used for hypocotyl elongation measurement were planted in the control plot and OTC setups (**Supplementary Figure S8**) and bolting and flowering time of the plants were scored. The aboveground temperature of the OTC^w+^ was 16.54 ± 0.05°C on average and exceeded the OTC^w−^ temperature by 4.07°C in the experimental period (**Supplementary Figure S10A**) between planting (4 March, 2024) and the moment the last plant opened its flowers (20 May, 2024). Similar effects were noted on soil and soil/air interface temperatures. Notably, the difference between OTC^w−^ and the empty control plot in the same period was 0.5°C (integral aboveground temperature, including the night period), indicating that passive warming can contribute to the overall temperature within the OTCs in (sunny) spring.

No obvious differences in relative survival (plants that made it to bolting) were noted between the plots across the genotypes, indicating that realistic field warming does not majorly contribute to Arabidopsis establishment and survival (**Supplementary Table S7**). Both genotype (*p <*1e−16) and (warming) treatment (*p <*1e−16) significantly explained variance in the moment of bolting. In total 20 accession bolted earlier in the OTC^w+^ setup compared with the empty control plot and 18 accessions bolted earlier in the OTC^w+^ compared with OTC^w−^ (**Supplementary Figure S11B**, **Supplementary Table S7**). Similarly, both genotype (*p <*1e−16) and (warming) treatment (*p <*1e−16) significantly explained variance in the moment the first flower opened. Overall, flowering occurred earlier in the OTC^w+^ setup compared with the empty control plot (accessions earlier) and 19 accessions flowered earlier in the OTC^w+^ compared with OTC^w−^ (**Figure 5C**, **Supplementary Table S7**). No major differences were noted in the absolute number of days between bolting and moment of flowering, although differences were significant (**Supplementary Table S7**; *p <*1e−5). Together, these data indicate that both active (OTC^w+^) and passive (OTC^w−^) field warming accelerates bolting and flowering in *Arabidopsis thaliana*. These findings indicate that our OTC setups can be used to reliably assess effects of mild high ambient temperature on *Arabidopsis thaliana* plant development and phenology in field conditions.

#### Tomato (*Solanum lycopersicum*)

Subsequently, we assessed the effect of field warming on tomato development and phenology. Tomato growth is optimal at relatively warm conditions (well above 20°C) (Adams et al., 2001). Tomato cv. Moneymaker seeds were therefore planted in late spring (2024) in pots and pre-germinated in an indoor growth chamber at 21°C. Once ~25% of the seeds had germinated, the pots were assigned to treatment groups and placed in the empty control plot and both OTCs. When the first true leaf became visible, the hypocotyl was measured and the plant was placed in pre-watered bare soil within the respective control plot or OTC (**Supplementary Figure S9**). On average, the temperature of the OTC^w+^ was 22.41 ± 0.27°C and exceeded the OTC^w−^ by 4.26°C (**Supplementary Figure S10A**) during the experimental period between transfer of the seedlings outside (May 8, 2024) and the moment the last plant exhibited the first visible true leaf (May 28, 2024). The integral difference between OTC^w−^ and the empty control plot was 0.86°C in this period, which can be attributed to passive warming effects. Similar differences were noted for soil and soil/air interface temperature. In total, 85.7% of seeds germinated in the OTC^w+^ plot, 84.31% in the OTC^w-^ plot and 67.31% in the empty control plot. The reason why the germination rates in the empty control plot are lower is unclear, but we deem this can be attributed by the lower temperature in the OTC^w−^ or differences in, *e.g.*, soil moisture. Although hypocotyls of OTC^w+^ plants were on average slightly longer compared with those in the OTC^w−^ setup and control plot, the differences were not significant (**Figure 5D**).

Field warming (OTC^w+^) had a more pronounced effect on vegetative plant growth and development. Five days after the transfer of the pots from the indoor to the outdoor environment, 18.8% of the seedlings in the OTC^w+^ had visible first leaves, whereas none of the plants in the empty control plot nor in the OTC^w−^ had reached that stage. After transfer to the wetted soil, we let the plants develop until the moment the first plants started to flower (period between May 14, 2024, and June 27, 2024). During this period, the average temperature of the OTC^w+^ was 21.75 ± 0.49°C, which exceeded the OTC^w−^ temperature by 4.28°C (**Supplementary Figure S10A**). The integral difference between OTC^w−^ and the empty control plot was 0.76°C in the experimental period, suggesting again that passive warming contributed to the OTC^w−^ temperature in this period. In the OTC^w+^, internodes of vegetative plants became significantly longer (**Figure 5E**) and more internodes (and thus leaves) formed (5.5 ± 1.46) compared with plants in the OTC^w−^ setup (4.42 ± 1.06) and control plot (4.48 ± 1.47). Altogether, this suggests that our OTC setup is suitable to study tomato growth and development in warmed field conditions.

## Discussion

We constructed and validated an Open Top Chamber (OTC) field warming setup with suspended heating cables that is able to generate 3 °C-5 °C of warming above the ambient temperature in the temperate sea climate of the Netherlands (**Figure 3**, **Supplementary Figure S10A**). This differs from an identical OTC^w−^ that is not actively warmed but can passively warm up slightly during sunny periods in spring and summer and from an empty control plot (**Supplementary Figure S10A**).

The level of warming achieved in the OTC^w+^ aligns well with the current middle to worst case global warming forecasts (Lee et al., 2023), which predict a rise of 0.4 °C-4°C in the coming decades. Hence, snowdrops (selected for their cold tolerance), tomato (having a high temperature requirement), and Arabidopsis (spring blooming model organism), placed in the actively warmed OTC^w+^ setup, display expected phenotypic and phenological traits attributable to the warmed condition. First, shoots of snowdrops in the OTC^w+^ were longer than those in the OTC^w−^ plot, which aligns observations that shoot lengths are up to 50% shorter when grown at −1°C compared with 6°C (Orthen and Wehrmeyer, 2004). Secondly, most Arabidopsis accessions displayed longer hypocotyls in the OTC^w+^ plot and exhibited earlier bolting and flowering, which respectively aligns with hypocotyl length being a hallmark trait of thermomorphogenesis induced by high temperatures (Quint et al., 2016; Ibañez et al., 2017; van der Woude et al., 2021; Delker et al., 2022) and observations that high temperatures induce earlier flowering (Balasubramanian et al., 2006). Finally, we found that OTC warming aids tomato development (sped up leaf formation, induced earlier flowering, and induced longer internodes), in line with previous observations (Adams et al., 2001).

### Advantages, limitations, and improvement of the OTC setup

In our semi-controlled OTC approach, the only empirically tweaked parameter differing between the OTC^w+^ and OTC^w−^ setups was active temperature input into the system by the heating cables. It is known from other field warming studies that heating can cause a decrease in plot moisture and OTCs can interfere with wind patterns (Ettinger et al., 2019). Additionally, OTC structures may impact rainfall along the perimeter of the plot, thus further interfering with some natural abiotic variables. However, our data on belowground soil moisture content indicated that at the depth of 10cm both OTCs retained similar amounts or even slightly more moisture than the empty control plot throughout the experimental period (**Supplementary Figure S10B**). Overall, differences in trait values between the OTC^W−^ and control plot were negligible, and the maximum observed difference in temperature throughout an experimental period was 0.86°C (**Supplementary Figure S10A**, period of tomato hypocotyl length assessment), which is likely attributable to the passive warming effect through the PMMA material used for the OTC and possibly aided by shelter from wind. This suggests that the physical structure of the OTC did not majorly impact the measured plant traits, at least not during the experimental period of this project (winter to late spring, 2024). Therefore, observed trait differences between OTC^w+^ and OTC^w−^ can be largely assigned to active warming.

We used regular transparent PMMA in our setup, which is only partially UV transparent. This was because UV-transparent PMMA is almost double the cost of regular PMMA and keeping costs low was one of the primary design goals. Because the wall of the OTC is angled by 60°, up to 40% of the ground area of the OTC falls in the UV shadow of the OTC wall at some point during the day. Thus, areas that are in relatively close proximity to the PMMA will have different UV exposure than the areas closer to the middle of the OTC. However, since plants are added to our setup, rather than having the OTC placed over existing vegetation, UV shadowing can be circumvented to a reasonable degree by avoiding planting close to the OTC PMMA walls, which we have put in practice in our current study (see **Supplementary Figures S8**, **S9**). Compared with Bokhorst et al. (2008) and Sun et al. (2013), our OTC was constructed with thicker PMMA. Therefore, no additional reinforcements were needed, which further cut down on material and manufacturing costs.

The distributed double (on the ground and suspended) heating cable spoke and wheel layout (layout #4) proved effective at increasing temperature throughout the OTC, and this layout leaves sufficient effective growth space for plants in reasonable proximity to the cables and in reasonable distance of the PMMA walls. However, our results also indicate that the OTC^w+^ requires continuous energy input as it does not retain heat well when not exposed to internal warming (**Figure 2**), given that passive warming effects are minimal (**Supplementary Figure S10A**). In addition, raw energy input is the most important factor in determining overall temperature increase in the OTC^w+^ (**Figure 1**, **Supplementary Figure S5**). These findings, taken together with Johnson et al. (2013) who reported that OTCs do not provide significant long-term heating, indicate that the primary purpose of the OTC is to provide shelter for the heating cable setup rather than being a significant driver of temperature increase themselves, although the structure likely adds to temperature uniformity within the OTC (Hollister et al., 2023).

Although the current setup proved very stable and effective in all weather conditions, several improvements and features could be implemented to aid future usage such as implementation of wireless data offloading options, real-time system analyses and remote control. In addition, temperature feedback regulation could be developed and measures could be taken to improve distribution/uniformity of temperature within the OTCs. Finally, additional sources of warming could be installed to further boost the temperature and uniformness. We discuss these proposed improvements in depth in **Supplementary File S1**.

## Conclusion

Our setup proved effective for persistently imposing a realistic mimic of global warming on plants, without significantly interfering with other dynamic environmental parameters. This differs from experiments in stable indoor climate rooms or cabinets. One notable advantage is that our OTC setup facilitates temperature experiments on plant species that require specific conditions, such as experiments on bulbous snowdrops (*Galanthus nivalis*), which need near-freezing temperatures for their development. These conditions are difficult to replicate in indoor plant growth facilities. Taken together, our setup is meant to facilitate experimental plant research and expands the range of plant growth facilities particularly in the context of field warming, although long-term performance and viability of the used materials remains to be tested. This is an important addition as actively warmed systems, such as ours, are relatively rare in the literature. Most documented OTCs have been deployed without additional heating into existing ecosystems (Bokhorst et al., 2008; Johnson et al., 2013). Our study should be considered a proof of concept as we only used two OTCs of which only one was actively warmed. However, the design of our OTC is flexible and can be easily scaled up in terms of OTC size and OTC numbers included in future (ecological) experiments. Although this would require some additional validations, this will undoubtedly further decrease the construction costs as well.

## Data Availability

The original contributions presented in the study are included in the article/**Supplementary Material**. Further inquiries can be directed to the corresponding author.

## Supplementary material

Supplementary Figure 1 Electronics layout within the weatherproofed plywood hutch, placed next to the OTC^w+^ (see **Figure 3**). Components are indicated by letters: **(A)** ESP8266 microcontroller, **(B)** Adalogger SD card data, **(C)** MOSFETs, **(D)** cooling fans, **(E)** 24V power supplies, **(F)** 230V sockets (right), **(G)** holes with fine mesh, **(H)** outdoor RCD powersocket, **(I)** fuses.

Supplementary Figure 2 Simplified circuit diagram of the electronics setup. Not pictured: 3V power supply to the microcontroller and AC power connected to the DC power supply.

Supplementary Figure 3
**(A)** Views of outdoor OTC plot establishment. **(A)** Empty plot, after weeding and adding nutrient-rich soil, before placement of OTCs. **(B)** empty control plot (left), OTC^w+^ with heating cables visible (middle) and OTC^w-^ (right). **(C)** TOMST microclimate logger placed in OTC^w+^. **(D)** Plywood hutch with electronics. Ventilation fan (blue bracket) and lock can be seen.

Supplementary Figure 4 Indoor OTC heating cable validation layouts. **(A-D)** Heating cables are the orange wires within the OTCs. Note the layer of potting soil that was placed inside the setup to aid soil temperature quantification. Indicated are: **(A)** layout #1, **(B)** layout #2, **(C)** layout #3A and **(D)** layout #4. Layout #3b is not shown here, but is present as the top layer of cables in layout #4 **(D)**. See **Supplementary Figure S5** for corresponding IR thermographs.

Supplementary Figure 5 IR thermographs obtained during indoor validation experiments of heating cable layouts. **(A)** empty control plot (no cables), **(B)** layout #1, **(C)** Layout #2, **(D)** Layout #3a, **(E)** Layout #3b and **(F)** Layout #4. Note that Layout #4 by far outperforms the other layouts in terms of plot heating. Scale bar next to each panel indicates the detected temperature range within the OTC per cable layout (°C).

Supplementary Figure 6 Indoor validation temperature dynamics of OTC without heating cables. **(A, B)** Temperature data measured inside the OTC placed indoors in the absence of heating cables. **(A)** OTC temperature measured with thermocouples at random spots in the OTC (thermocouple probes #1-3; indicated in red-to-orange) and two thermocouples placed outside of the OTC (#4-5; indicated in yellow and blue), representing the (baseline) test room temperature. **(B)** Soil temperatures as obtained by IR thermography. Spot measurements were taken from the overall thermal picture capturing the whole OTC, in the center of the OTC, and four spots perpendicular to each other in between the center (brown) and the edge of the OTC, indicated as down (blue), left (purple), right (pink) and up (light blue).

Supplementary Figure 7 Thermal profiles of the OTCs and empty control plot. **(A)** Overview of the IR thermography setup used to capture the thermal images of panel B-E. **(B)** Thermal overview of the outdoor site. On the foreground the empty control plot (no OTC) and plywood hutch with electronics, in the middle the OTC^w+^ and the OTC^w-^ can be seen in the background. **(C-E)** IR thermography images of **(C)** the control plot (no OTC), **(D)** the OTC^w-^ and **(E)** the OTC^w+^ setup. Note the warm heating cables running in a spoke wheel pattern in the OTC^w+^ **(D)** and the passive warming effect present in the OTC^w-^ **(E)**. Also note the trays containing Arabidopsis plants for phenology assessment (flowering time and bolting time). Images were taken on March 24, 2024 on a sunny spring day. Scale bar next to each panel indicates the detected temperature ranges in panels **(B-E)** (°C).

Supplementary Figure 8 Arabidopsis hypocotyl length and phenology assessment. **(A-C)** Top view images of **(A)** the empty control plot without OTC, **(B)** the OTC^w-^ and **(C)** the OTC^w+^ setups. In all panels vertically-oriented MS-Agar plates containing seedlings used for hypocotyl assays and the TOMST microclimate dataloggers can be seen, as well as the trays containing the different natural accessions (*Arabidopsis thaliana* plants contained in racks) used for assessing plant phenology (bolting time and flowering time).

Supplementary Figure 9 Assessment of tomato development. **(A, B)** Top view images of tomato planted in the OTC^w+^ setup.

Supplementary Figure 10 TOMST microclimate logger readouts during trait and phenology assessment periods of snowdrop, Arabidopsis and tomato. **(A)** Average temperatures (T, °C) 10cm above ground (left column), 10cm below ground (middle column), at the soil/air interface (right column) and **(B)** Soil moisture content 10cm belowground during the experimental periods. Indicated are: The period between *Galanhus nivalis* Niënhof emergence and shoots harvest (January 22, 2024 - February 13, 2024; upper row; Snowdrop), during the Arabidopsis hypocotyl length experiment (March 26, 2024 - April 2, 2024; Second row; Ara Hyp Len), during Arabidopsis bolting and flowering time assessment (March 4, 2024 – May 20, 2024; third row; Ara Phenology), during tomato seedling hypocotyl measurements (May 8, 2024 – May 28, 2024; fourth row, Tom Hyp Len) and during tomato vegetive development (May 14, 2024 - June 27, 2024; bottom row; Tom Internode). In each panel the values of both sensors per OTC/empty plot are indicated. Sensors in the empty control plot **(C)** are indicated in grey, the OTC^w-^ plot in blue and the OTC^w+^ in red.

Supplementary Figure 11 Hypocotyl elongation of *Arabidopsis thaliana* plants cultivated in climate-controlled cabinets and effect of OTC field warming on days to bolting. **(A)** Hypocotyl lengths of 8 day-old seedlings grown in climate-controlled cabinets set at 10 °C (blue boxes) or 20 °C (red boxes) under a 10-hour photoperiod (n = 3–23; median n = 20, significant differences at *p <*0.05 (unpaired t-test) are indicated with an asterisk. When no asterisk is shown, the difference was not significant). **(B)** numbers of days until the moment the bolt appeared (n = 1–11; median n = 7; ANOVA: genotype p < 1e-16, treatment: p < 1e-16, interaction p = 0.48) of Iberian peninsula and locally-collected *Arabidopsis thaliana* accessions, placed in the OTC^w-^ (blue), OTC^w+^ setup (red) or the empty control plot (grey). Boxes indicate boundaries of the second and third quartiles (Q) of the data distribution. Black horizontal bars indicate median and whiskers Q1 and Q4 values within 1.5 times the interquartile range. Dots represent outliers beyond Q1 and Q4.
