# Supplementary material for: Design and construction of a low-cost, low-input Open Top Chamber field warming setup to assess aboveground plant response to global warming
Source: Front Plant Sci. 2025 Oct 14;16:1677291. doi: 10.3389/fpls.2025.1677291 (PMC12560058; doi:10.3389/fpls.2025.1677291)
Supplement: Supplementary Figure 1 — Electronics layout within the weatherproofed plywood hutch, placed next to the OTCw+ (see Figure 3 ). Components are indicated by letters: (A) ESP8266 microcontroller, (B) Adalogger SD card data, (C) MOSFETs, (D) cooling fans, (E) 24V power supplies, (F) 230V sockets (right), (G) holes with fine mesh, (H) outdoor RCD powersocket, (I) fuses. [file SupplementaryFile1.zip › Supplementary Table 3.PDF]

Supplementary Table S3. Overview of indoor validation experiments. Start time is the time at which the temperature recording equipment and the heating cables were turned on. Cables Off is the time at which the heating cables were turned off. End time is the time at which the data were collected and the run was over.

Cable layout	Date of experiment	Start	Cables Off	End
No cables	21-4-2023 – 24-4-2023	14:50	NA	14:50
Layout #1	11-5-23	10:25	16:35	18:30
Layout #2	23-5-23	11:15	15:30	18:30
Layout #3a	1-6-23	13:10	16:05	18:15
Layout #3b	6-6-23	12:10	15:30	17:00
Layout #4	9-6-23	10:55	14:00	16:30
