# Supplementary material for: Design and construction of a low-cost, low-input Open Top Chamber field warming setup to assess aboveground plant response to global warming
Source: Front Plant Sci. 2025 Oct 14;16:1677291. doi: 10.3389/fpls.2025.1677291 (PMC12560058; doi:10.3389/fpls.2025.1677291)
Supplement: Supplementary Figure 1 — Electronics layout within the weatherproofed plywood hutch, placed next to the OTCw+ (see Figure 3 ). Components are indicated by letters: (A) ESP8266 microcontroller, (B) Adalogger SD card data, (C) MOSFETs, (D) cooling fans, (E) 24V power supplies, (F) 230V sockets (right), (G) holes with fine mesh, (H) outdoor RCD powersocket, (I) fuses. [file SupplementaryFile1.zip › Supplementary Table 1.PDF]

Supplementary Table S1. Overview of field warming experiment types and there (dis)advantages. Typically, existing field warming setups can be divided into distinct groups based on the type of heating device used and the direct target of warming. Example papers are provided.

**Heating Type**	**Target**	**Advantages**	**Disadvantages**	**Notable Papers**
Ceramic Infra-red Heaters	Surfaces (plant +soil)	Excellent recovery time, good heat distribution, minimally effected by weather changes. Most commonly implement in literature.	Energy intensive. Does not heat soil well. Can vary in efficacy based on type of plants.	(Han et al., 2014; Kimball et al., 2008)
Open Top Chambers (OTC)	Air	Passive, no power requirement. Cheap to build and implement. Minimal environmental disturbance	No active control. Interferes with wind and potentially other natural variables. Highly weather dependent. High variability in effectiveness	(Bokhorst et al., 2008; Sun et al., 2013)
Forced air heaters	Air	Good recovery time. Easier to monitor than Infra-red heaters.	Energy intensive. Requires more expensive infra-structure up front. Must be coupled with OTC to be effective. Interfere with environmental variables.	(Hanson et al., 2017)
Heating cables, buried	Soil	Very efficient.	Potential disruptive to plots. Can create significant temperature gradients	(Hanson et al., 2011; Patil et al., 2013)
Heating cables, surface	Soil surface + air	Reasonably efficient, minimally disruptive, cheap.	Slow response time. Creates heat gradients.	(O'Neill et al., 2019)
