# Supplementary material for: Design and construction of a low-cost, low-input Open Top Chamber field warming setup to assess aboveground plant response to global warming
Source: Front Plant Sci. 2025 Oct 14;16:1677291. doi: 10.3389/fpls.2025.1677291 (PMC12560058; doi:10.3389/fpls.2025.1677291)
Supplement: Supplementary Figure 1 — Electronics layout within the weatherproofed plywood hutch, placed next to the OTCw+ (see Figure 3 ). Components are indicated by letters: (A) ESP8266 microcontroller, (B) Adalogger SD card data, (C) MOSFETs, (D) cooling fans, (E) 24V power supplies, (F) 230V sockets (right), (G) holes with fine mesh, (H) outdoor RCD powersocket, (I) fuses. [file SupplementaryFile1.zip › Supplementary Table 2.PDF]

Supplementary Table S2. List of materials and electronics components used to construct the OTC setups, with manufacturer name and parts numbers.

Material Description	Material Name	Manufacturer	Location	Parts Number/Product ID	Technical Specs
5 mm PMMA sheet	Altuglas CN PMMA	Altuglas International	Paris, France	Altuglas CN Clear 100 10000	Cast, 5mm thick
Heating cables	NA	Decochip	Netherlands	7423418520541	18W/m
Power supply	NA	Mean Well	USA	LRS-350-24	AC/DC converter 24V 350W
GFCI	safety adapter	REV Ritter	Mömbris, Germany	4008297154945	230V, 30mA, IP44
Microcontroller	Adafruit Feather HUZZAH with ESP8266	Adafruit	New York, NY, USA	2821	NA
MOSFET	NA	Infineon	Germany	IRLZ34NPbF	NA
SD logger + RTC	Adalogger FeatherWing - RTC + SD	Adafruit	New York, NY, USA	2922	NA
Thermocouple Amplifier	Adafruit MCP9600 I2C Thermocouple Amplifier - K, J, T, N, S, E, B and R Type T	Adafruit	New York, NY, USA	4101	NA
Type T Thermocouple	NA	Labfacility	Dinnington, UK	Z2-T-2M (IEC)	NA
Multicore cable	NA	Multicomp Pro	NA	3183Y-1.50MMWHT	3 core, 1.5mm^2, unscreened
6 core ribbon cable	NA	Pro Power	NA	R2651DTSY06SC85	28 AWG, unscreened
Cooling fans	NA	Sinwan	Taipei, Taiwan	S938AP-22-1	230V AC
Computer fan	NA	Multicomp	NA	MC001581	24V DC
Screw terminal block	NA	Amphenol Anytek	NA	TJ045153000AG	4 wire
Screw terminal header	NA	Amphenol Anytek	NA	OQ045450000AG	4 wire
IDC connector	NA	Amphenol	NA	T812106A100CEU	6 wire
Pin header	NA	Amphenol	NA	T821106A1R100CEU	6 wire
