# Supplementary material for: Design and construction of a low-cost, low-input Open Top Chamber field warming setup to assess aboveground plant response to global warming
Source: Front Plant Sci. 2025 Oct 14;16:1677291. doi: 10.3389/fpls.2025.1677291 (PMC12560058; doi:10.3389/fpls.2025.1677291)
Supplement: Supplementary Figure 1 — Electronics layout within the weatherproofed plywood hutch, placed next to the OTCw+ (see Figure 3 ). Components are indicated by letters: (A) ESP8266 microcontroller, (B) Adalogger SD card data, (C) MOSFETs, (D) cooling fans, (E) 24V power supplies, (F) 230V sockets (right), (G) holes with fine mesh, (H) outdoor RCD powersocket, (I) fuses. [file SupplementaryFile1.zip › Supplementary Table 7.PDF]

Supplementary Table S7. Survival and phenology of natural accessions planted in the OTCs and control plot. 11 Iberian peninsula collection accessions (abbreviation with letters and number) and 10 locally collected accessions (coded with C#C; See Supplementary Table S4) were planted in the empty control plot (C), OTC^{w-} (W-) or OTC^{w+} (W+). Indicated are the total (absolute) number of plants that established and survived until flowering, the relative contribution per genotype to the total number of established flowering plants, the total (absolute) number of days until the bolting stem became visible, total (absolute) number days to the first flower opened and total (absolute) number of days between the moment the bolting stem became visible and the moment the first flower opened. White-to-green shading indicates the value distribution per trait, with low values indicated in white and highest values indicated in dark green.

		number surviving			relative contribution			days to bolting			days to flower open			days beteen bolting and flower open		
		C (empty)	OTCW-	OTCW+	C (empty)	OTCW-	OTCW+	C (empty)	OTCW-	OTCW+	C (empty)	OTCW-	OTCW+	C (empty)	OTCW-	OTCW+
Iberian penisula collection	Ala-0	9	6	4	6,8%	4,0%	3,6%	63,9	62,2	53,0	72,0	70,2	59,3	8,1	8,0	6,3
	Ale-4	4	3	2	3,0%	2,0%	1,8%	73,8	76,3	69,0	83,8	80,3	74,0	10,0	4,0	5,0
	Bea-0	7	10	9	5,3%	6,7%	8,0%	63,6	60,0	59,4	72,0	68,6	62,8	8,4	8,6	3,5
	Bis-0	1	3	1	0,8%	2,0%	0,9%	84,0	84,3	82,0	91,0	91,3	89,0	7,0	7,0	7,0
	Bus-0	10	10	5	7,5%	6,7%	4,5%	67,9	64,7	61,6	76,2	72,9	69,4	8,3	8,2	7,8
	Cap-1	3	3	2	2,3%	2,0%	1,8%	71,7	71,0	72,5	76,3	76,3	76,0	4,7	5,3	3,5
	Doñ-0	2	5	1	1,5%	3,3%	0,9%	72,5	65,8	59,0	76,0	72,8	61,0	3,5	7,0	2,0
	fei-0	7	8	8	5,3%	5,3%	7,1%	62,9	60,0	55,3	72,1	66,0	58,9	9,3	6,0	3,6
	Gra-0	3	4	1	2,3%	2,7%	0,9%	87,0	87,0	84,0	91,0	91,3	91,0	4,0	4,3	7,0
	Pig-0	9	8	9	6,8%	5,3%	8,0%	63,2	59,8	54,0	66,8	62,1	57,2	3,6	2,4	3,2
	Rab-7	8	6	6	6,0%	4,0%	5,4%	66,1	63,7	61,5	76,0	73,3	67,3	9,3	9,7	5,8
Local accessions	C117c	5	9	3	3,8%	6,0%	2,7%	70,2	62,9	63,3	79,4	74,1	66,7	9,2	11,2	3,3
	C123c	8	6	5	6,0%	4,0%	4,5%	75,8	70,8	66,4	81,4	75,0	76,0	5,6	4,2	7,3
	C165c	9	11	9	6,8%	7,3%	8,0%	75,0	75,5	74,3	83,6	82,8	79,9	8,6	6,7	5,6
	C171c	8	10	9	6,0%	6,7%	8,0%	76,9	73,4	68,3	83,4	80,4	78,3	6,5	7,0	9,6
	C173c	8	9	7	6,0%	6,0%	6,3%	76,8	69,6	66,4	82,4	78,1	77,0	5,4	8,6	10,6
	C175c	5	9	8	3,8%	6,0%	7,1%	84,8	77,4	81,4	89,0	83,7	86,3	4,2	6,2	5,3
	C178c	8	8	9	6,0%	5,3%	8,0%	67,3	63,1	60,8	75,0	73,8	67,8	9,6	10,6	7,0
	C180c	4	5	3	3,0%	3,3%	2,7%	78,5	75,0	71,0	84,8	82,8	77,0	6,3	7,8	9,0
	C199c	7	7	7	5,3%	4,7%	6,3%	79,0	75,0	71,1	82,3	79,6	74,7	4,2	4,6	5,7
	C217c	8	10	4	6,0%	6,7%	3,6%	82,0	77,6	79,5	87,8	86,4	84,8	5,8	8,8	5,3
