# Supplementary material for: Design and construction of a low-cost, low-input Open Top Chamber field warming setup to assess aboveground plant response to global warming
Source: Front Plant Sci. 2025 Oct 14;16:1677291. doi: 10.3389/fpls.2025.1677291 (PMC12560058; doi:10.3389/fpls.2025.1677291)
Supplement: Supplementary Figure 1 — Electronics layout within the weatherproofed plywood hutch, placed next to the OTCw+ (see Figure 3 ). Components are indicated by letters: (A) ESP8266 microcontroller, (B) Adalogger SD card data, (C) MOSFETs, (D) cooling fans, (E) 24V power supplies, (F) 230V sockets (right), (G) holes with fine mesh, (H) outdoor RCD powersocket, (I) fuses. [file SupplementaryFile1.zip › Supplementary Table 5.PDF]

Supplementary Table S5. Air temperature summary data of indoor validation experiments, as measured using thermocouples, from the 5 tested cable layouts and no cable experiments. Data is shown °C. Data are a subset of the total experimental data using only the data points from the heated portion of each experiment. Data points from all thermocouples placed inside the OTC in each experiment are combined to form one set of temperature data for each experiment.

Cable Layout	Min	1st Q	Median	Mean	3rd Q	Max
No Cables	17.38	18.12	18.31	18.38	18.69	19.38
Layout #1	20.38	20.75	21.38	21.21	21.56	21.81
Layout #2	20.88	22.31	22.69	22.73	23.19	24.56
Layout #3a	18.62	20.50	21.31	21.19	21.77	23.50
Layout #3b	20.75	21.50	21.69	21.71	21.88	22.94
Layout #4	22.19	24.06	24.56	24.53	25.06	27.75
