# Supplementary material for: Design and construction of a low-cost, low-input Open Top Chamber field warming setup to assess aboveground plant response to global warming
Source: Front Plant Sci. 2025 Oct 14;16:1677291. doi: 10.3389/fpls.2025.1677291 (PMC12560058; doi:10.3389/fpls.2025.1677291)
Supplement: Supplementary Figure 1 — Electronics layout within the weatherproofed plywood hutch, placed next to the OTCw+ (see Figure 3 ). Components are indicated by letters: (A) ESP8266 microcontroller, (B) Adalogger SD card data, (C) MOSFETs, (D) cooling fans, (E) 24V power supplies, (F) 230V sockets (right), (G) holes with fine mesh, (H) outdoor RCD powersocket, (I) fuses. [file SupplementaryFile1.zip › Supplementary Table 4.PDF]

Supplementary table S4. Collection sites of local accessions. Indicated are Accession ID (coded with C#C), Latitude, Longitude and the municipality and neighborhood/estate name of the collection site.

Accession ID	Latidude	Longitude	Location
C117c	52.06998	5.20502	Bunnik, Niënhof estate
C123c	52.07198	5.119907	Utrecht, Hoograven
C165c	52.06725	5.16927	Bunnik, Amelisweerd estate
C171c	52.07216	5.134572	Utrecht, Bokkenbuurt
C173c	52.05284	5.149881	Houten, 't Hemeltje
C175c	52.04472	5.175019	Houten, Nieuw Wulven estate
C178c	52.05816	5.129269	Utrecht, Lunetten
C180c	52.07407	5.121106	Utrecht, Rotsoord
C199c	52.03836	5.668797	Ede
C217c	52.07560	5.141029	Utrecht, Galgenwaard
