# Supplementary material for: Design and construction of a low-cost, low-input Open Top Chamber field warming setup to assess aboveground plant response to global warming
Source: Front Plant Sci. 2025 Oct 14;16:1677291. doi: 10.3389/fpls.2025.1677291 (PMC12560058; doi:10.3389/fpls.2025.1677291)
Supplement: Supplementary Figure 1 — Electronics layout within the weatherproofed plywood hutch, placed next to the OTCw+ (see Figure 3 ). Components are indicated by letters: (A) ESP8266 microcontroller, (B) Adalogger SD card data, (C) MOSFETs, (D) cooling fans, (E) 24V power supplies, (F) 230V sockets (right), (G) holes with fine mesh, (H) outdoor RCD powersocket, (I) fuses. [file SupplementaryFile1.zip › Supplementary File 1.DOCX]

**Supplemental Text S1**

**Future improvements of the Open Top Chamber field warming setup**

Although the current setup proved very stable and effective in all weather conditions, several improvements and features could be implemented to aid future usage. In the current setup, data retrieval options to extract logged data or monitor the system while it is running, either on location or remotely are underdeveloped. Currently the data collected by the system is stored on local micro-SD cards that must be manually removed from the data loggers in order to be read out. This is difficult for the untrained user and can lead to unexpected system errors or damage. Additionally, taking the card in and out could cause the data logger system to stop recording data or otherwise malfunction through no direct user error. To solve this, a separate data offloading function could be added. Qualitatively checking if the system properly heats is relatively easy as one can simply manually sense that the OTC inside and the cables are warm. If one, however, wants quantitative readings or to adjust the system beyond turning a group of cables on or off, one must connect the microcontroller to a computer and upload new code. This requires knowledge about coding and the specifics of this system. Second, it requires disconnecting the system to run diagnostics, which can be disruptive to experiments. A solution would be to include a small lcd screen that could provide system readouts on command. Additionally, these readouts could be tied into the Wi-Fi connection so that readouts could be collected and setting adjusted remotely. For instance, one could program different day and night settings, such that for instance only in the night the system warms. Wi-Fi connection would also allow for the system to monitor itself and alert the user of any issues.

A major functional feature addition would be to implement feedback regulation. Currently the cable groups are either on or off. However, this system would readily allow for power modulation by adjusting the MOSFET duty cycle. By adding some thermocouples in the plots, similar to those we used in the indoor experiments, a temperature feedback system could be constructed. The sophistication of this system could be of various levels from a basic feedback loop to a PID system wherein the system would predict changes in temperature and anticipate on the response time of the cables and adjust accordingly. The current configuration of the microcontroller also only allows for the cables to be controlled as two units. However, if the microcontroller was upgraded to one with more I/O pins, each cable could be controlled individually, potentially increasing the complexity of the feedback regulation to one of tailored sectional control. Feedback regulation would dramatically increase the usefulness of this setup because it would allow for nuanced temperature regulation rather than brute force, always on, warming.

Another potential system improvement would be to add an additional source of warming to the OTC to further boost the temperature and uniformness. This could be in the form of adding more heating cables (below or above ground), IR heaters, forced air, or something else. The heating cables are effective, but they are not perfect. In the coldest and warmest periods of the year, it may be necessary to increase the energy input to maintain a constant temperature increase above the prevailing ambient baseline. Currently, our setup has not been tested under severe freezing or heat. Typically field warming setups fall short of this (Ettinger et al., 2019), but the combination of more than one heating technique could potentially help. Another current limitation of our study is the absence of assessment of heat distribution in the OTC with cable layout #4 in field conditions. We suggest that heat distribution could be improved with the addition of forced air. This forced air could be provided by just a fan blowing air in a spiral manner within the OTC. At this basic level, the air current within the OTC would serve to distribute the hottest air coming off the heating cables and would also potentially serve to disrupt any vertical thermal gradients that may form within the OTC. In an ideal forced air setup, the ventilation would be connected to a heating element that could turn on and off depending on the energy input needs of the system. In addition, a forced air system could be coupled to a (de)moisturizer, allowing control and maintenance of relative humidity in the OTC space. It goes without mentioning that all improvements would have to be extensively tested including gathering higher resolution spatial temperature data of thermal gradients within the three-dimensional OTC space.
