# Supplementary figures and images for: Design and construction of a low-cost, low-input Open Top Chamber field warming setup to assess aboveground plant response to global warming
Source: Front Plant Sci. 2025 Oct 14;16:1677291. doi: 10.3389/fpls.2025.1677291 (PMC12560058; doi:10.3389/fpls.2025.1677291)

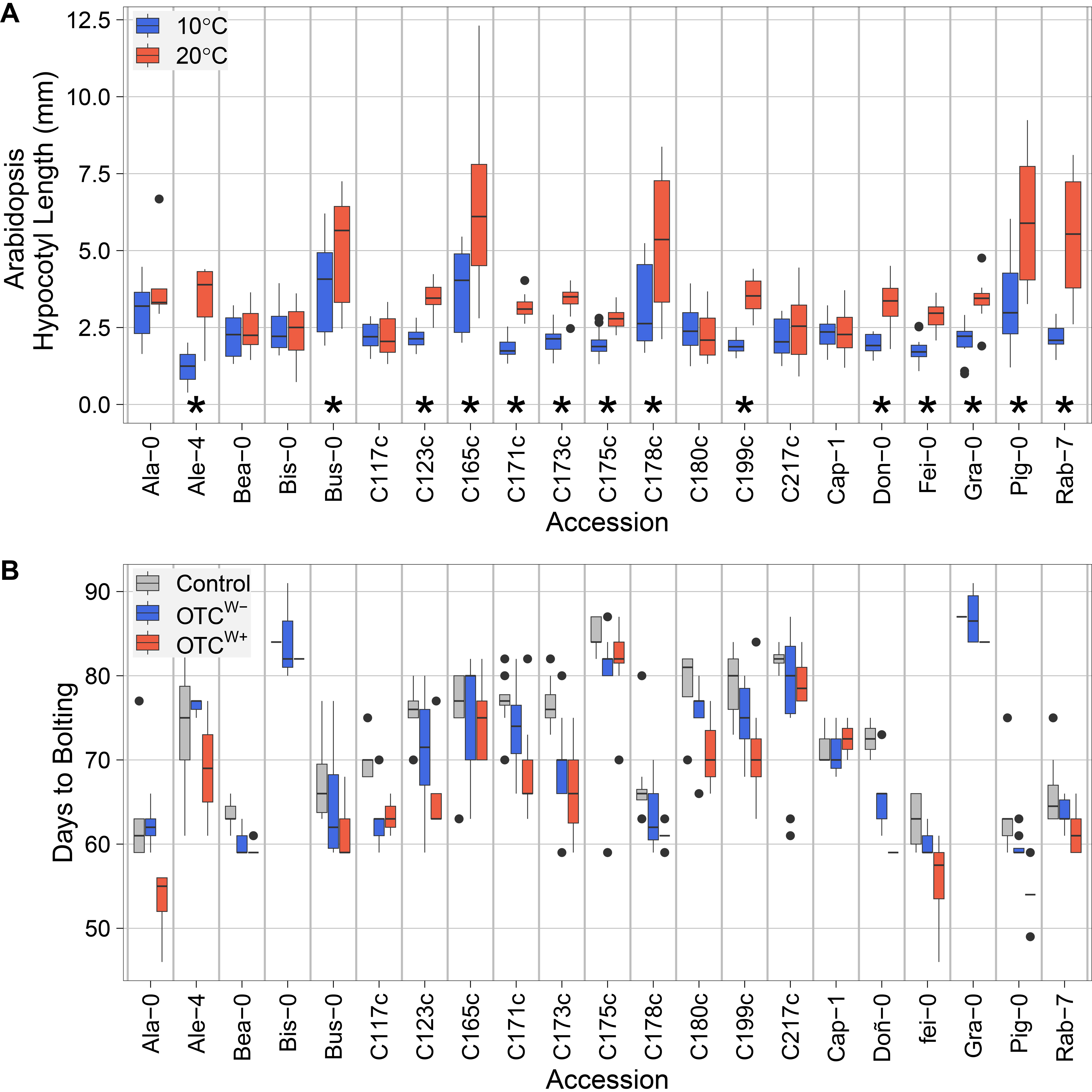

Supplement: Supplementary Figure 1 — Electronics layout within the weatherproofed plywood hutch, placed next to the OTCw+ (see Figure 3 ). Components are indicated by letters: (A) ESP8266 microcontroller, (B) Adalogger SD card data, (C) MOSFETs, (D) cooling fans, (E) 24V power supplies, (F) 230V sockets (right), (G) holes with fine mesh, (H) outdoor RCD powersocket, (I) fuses. [file SupplementaryFile1.zip › Supplementary Figure 11.JPEG]

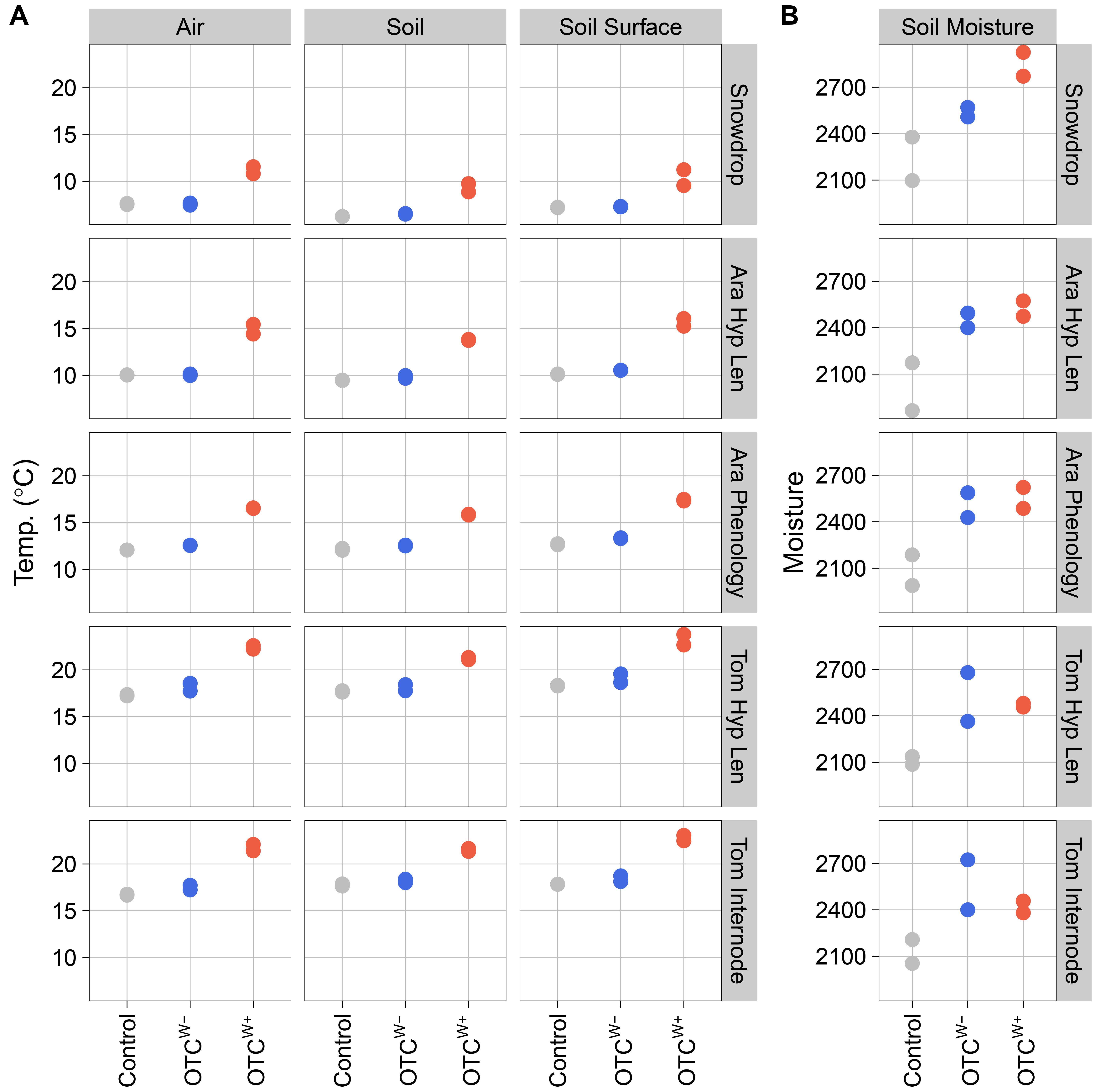

Supplement: Supplementary Figure 1 — Electronics layout within the weatherproofed plywood hutch, placed next to the OTCw+ (see Figure 3 ). Components are indicated by letters: (A) ESP8266 microcontroller, (B) Adalogger SD card data, (C) MOSFETs, (D) cooling fans, (E) 24V power supplies, (F) 230V sockets (right), (G) holes with fine mesh, (H) outdoor RCD powersocket, (I) fuses. [file SupplementaryFile1.zip › Supplementary Figure 10.JPEG]

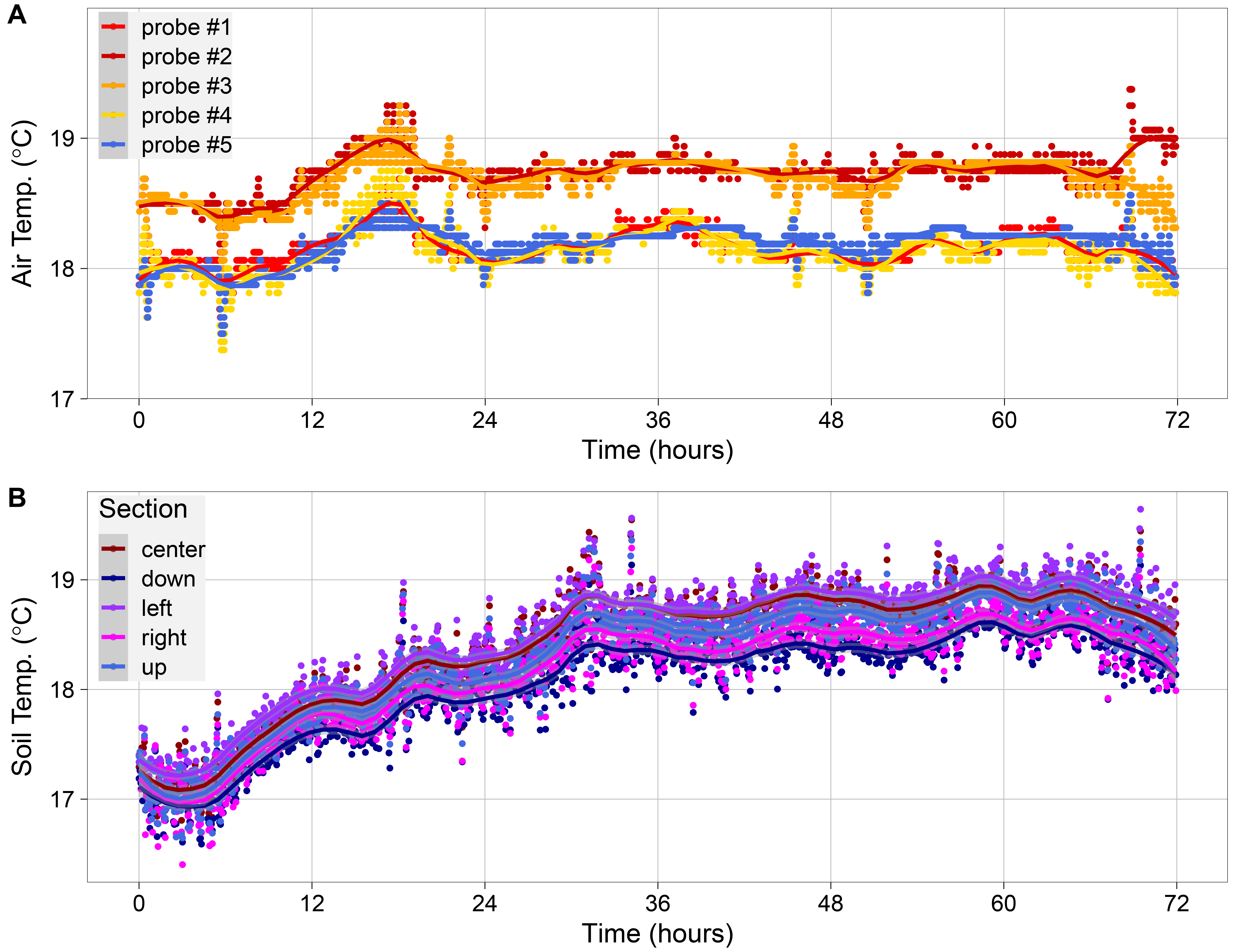

Supplement: Supplementary Figure 1 — Electronics layout within the weatherproofed plywood hutch, placed next to the OTCw+ (see Figure 3 ). Components are indicated by letters: (A) ESP8266 microcontroller, (B) Adalogger SD card data, (C) MOSFETs, (D) cooling fans, (E) 24V power supplies, (F) 230V sockets (right), (G) holes with fine mesh, (H) outdoor RCD powersocket, (I) fuses. [file SupplementaryFile1.zip › Supplementary Figure 6.JPEG]

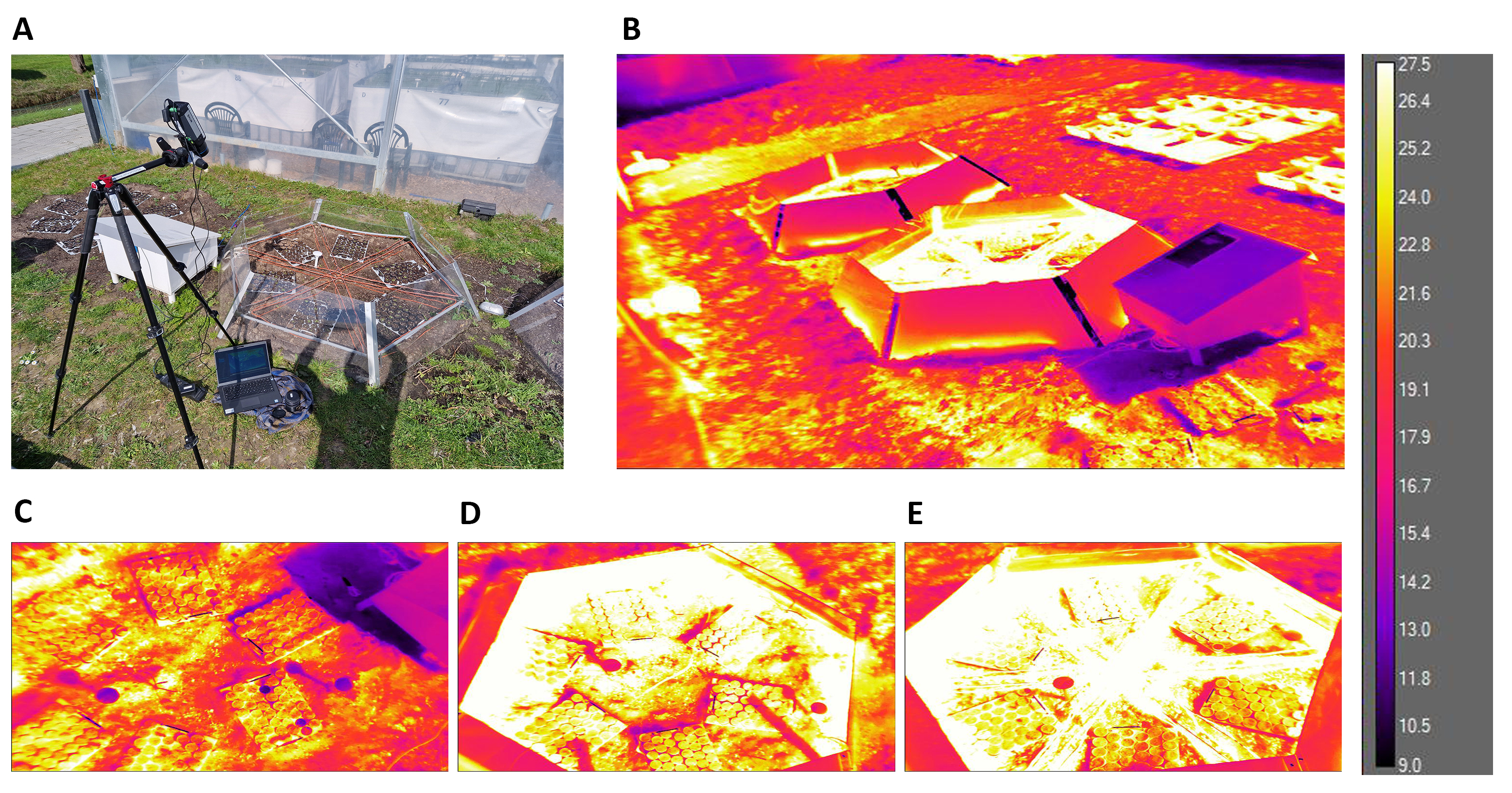

Supplement: Supplementary Figure 1 — Electronics layout within the weatherproofed plywood hutch, placed next to the OTCw+ (see Figure 3 ). Components are indicated by letters: (A) ESP8266 microcontroller, (B) Adalogger SD card data, (C) MOSFETs, (D) cooling fans, (E) 24V power supplies, (F) 230V sockets (right), (G) holes with fine mesh, (H) outdoor RCD powersocket, (I) fuses. [file SupplementaryFile1.zip › Supplementary Figure 7.JPEG]

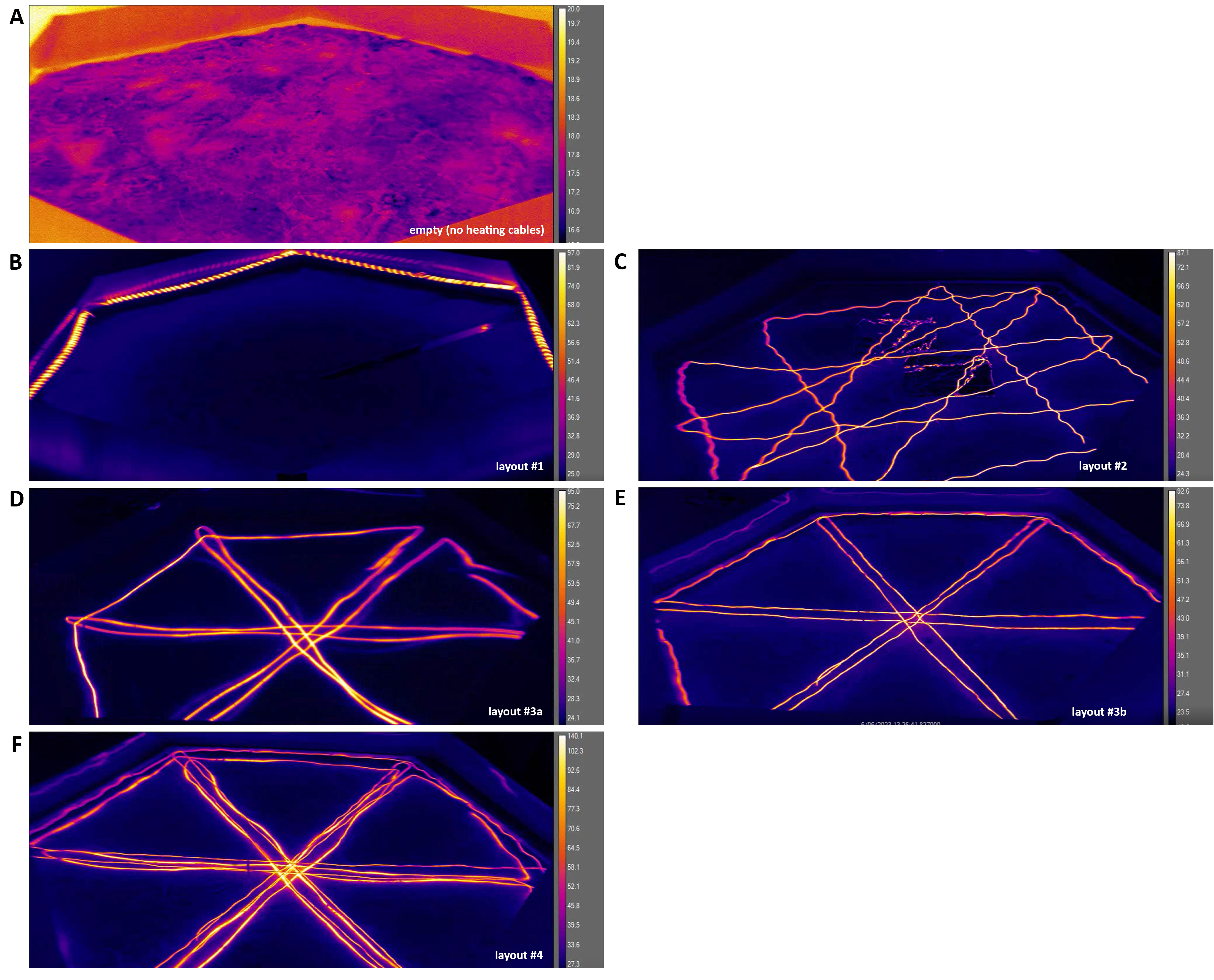

Supplement: Supplementary Figure 1 — Electronics layout within the weatherproofed plywood hutch, placed next to the OTCw+ (see Figure 3 ). Components are indicated by letters: (A) ESP8266 microcontroller, (B) Adalogger SD card data, (C) MOSFETs, (D) cooling fans, (E) 24V power supplies, (F) 230V sockets (right), (G) holes with fine mesh, (H) outdoor RCD powersocket, (I) fuses. [file SupplementaryFile1.zip › Supplementary Figure 5.JPEG]

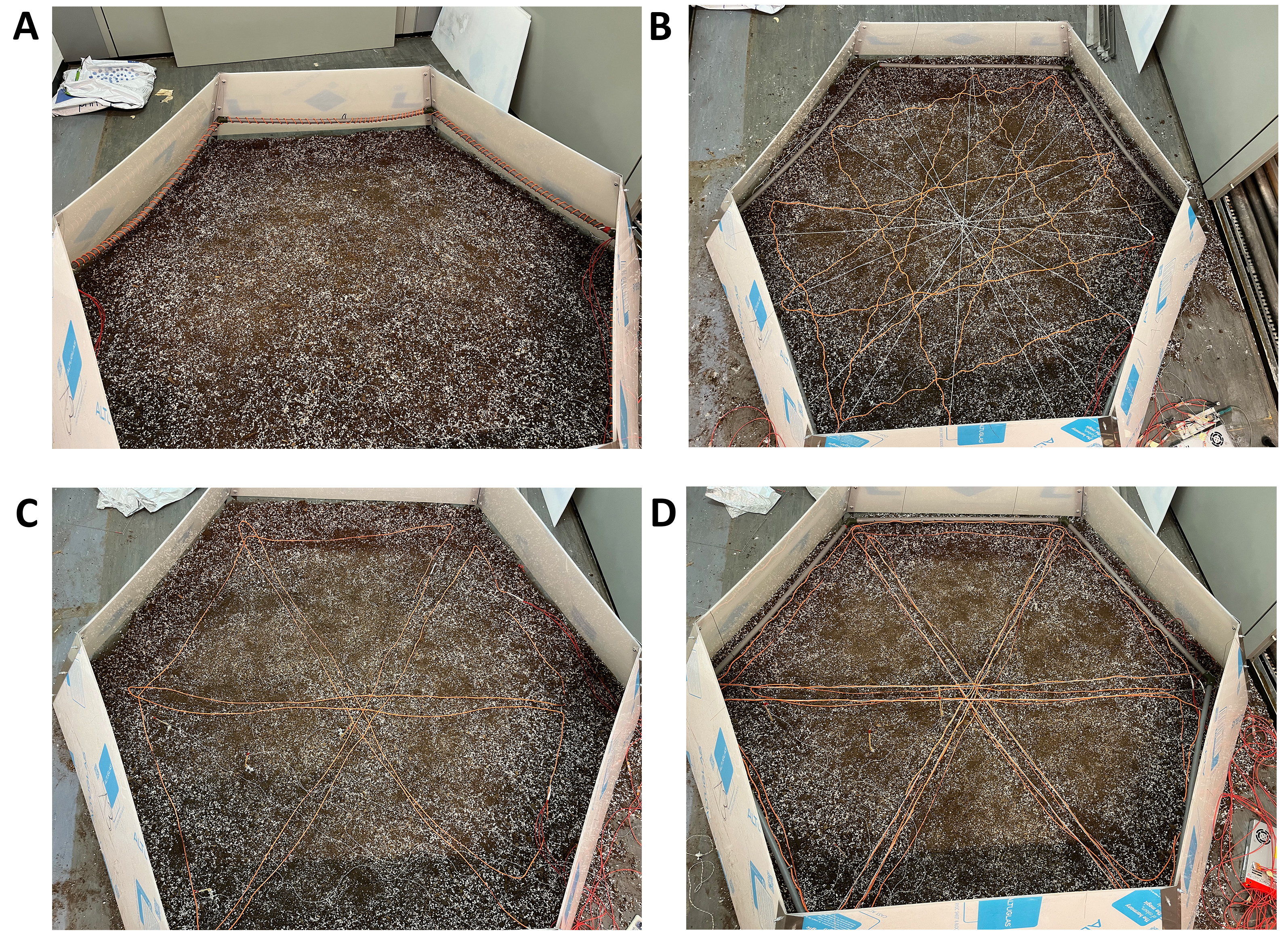

Supplement: Supplementary Figure 1 — Electronics layout within the weatherproofed plywood hutch, placed next to the OTCw+ (see Figure 3 ). Components are indicated by letters: (A) ESP8266 microcontroller, (B) Adalogger SD card data, (C) MOSFETs, (D) cooling fans, (E) 24V power supplies, (F) 230V sockets (right), (G) holes with fine mesh, (H) outdoor RCD powersocket, (I) fuses. [file SupplementaryFile1.zip › Supplementary Figure 4.JPEG]

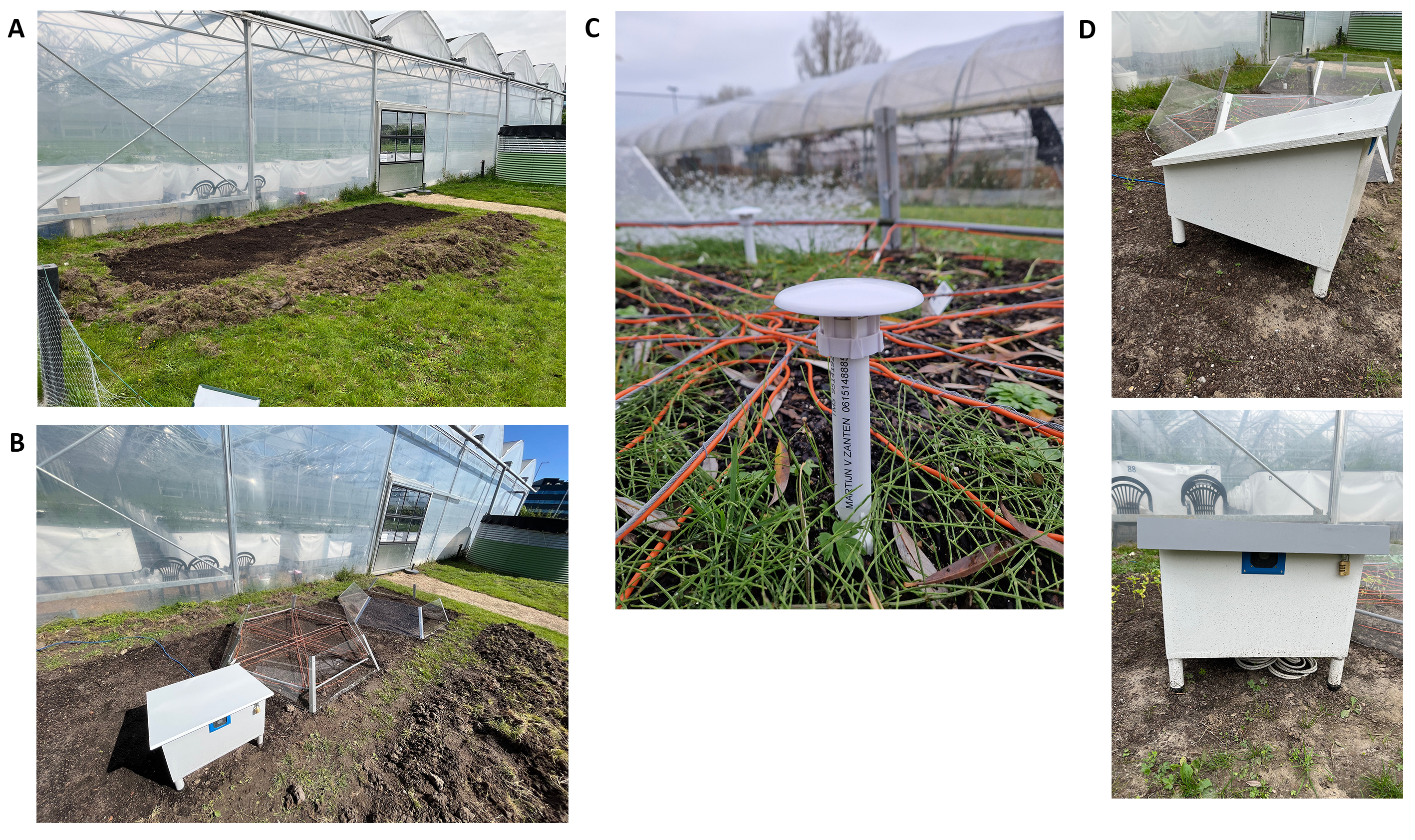

Supplement: Supplementary Figure 1 — Electronics layout within the weatherproofed plywood hutch, placed next to the OTCw+ (see Figure 3 ). Components are indicated by letters: (A) ESP8266 microcontroller, (B) Adalogger SD card data, (C) MOSFETs, (D) cooling fans, (E) 24V power supplies, (F) 230V sockets (right), (G) holes with fine mesh, (H) outdoor RCD powersocket, (I) fuses. [file SupplementaryFile1.zip › Supplementary Figure 3.JPEG]

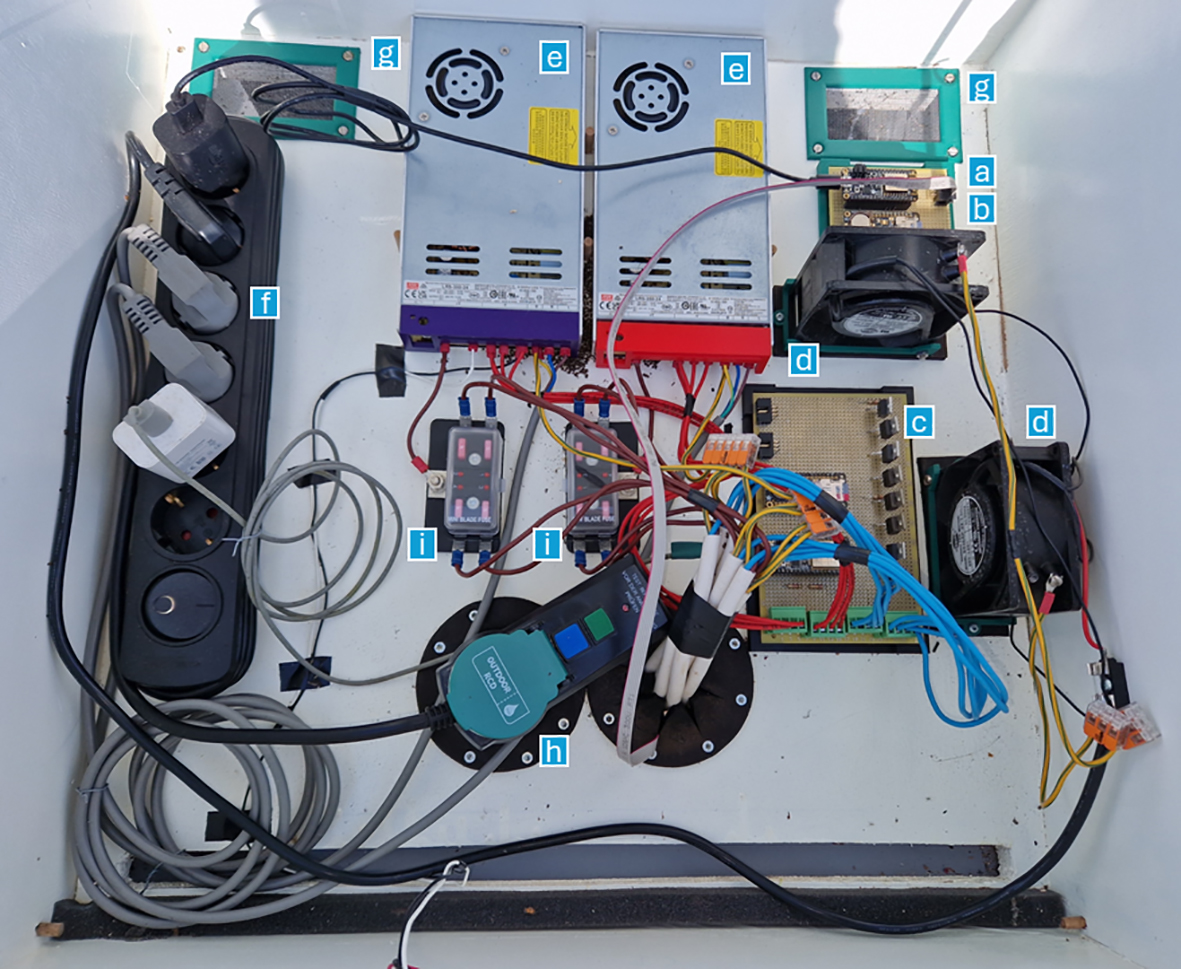

Supplement: Supplementary Figure 1 — Electronics layout within the weatherproofed plywood hutch, placed next to the OTCw+ (see Figure 3 ). Components are indicated by letters: (A) ESP8266 microcontroller, (B) Adalogger SD card data, (C) MOSFETs, (D) cooling fans, (E) 24V power supplies, (F) 230V sockets (right), (G) holes with fine mesh, (H) outdoor RCD powersocket, (I) fuses. [file SupplementaryFile1.zip › Supplementary Figure 1.JPEG]

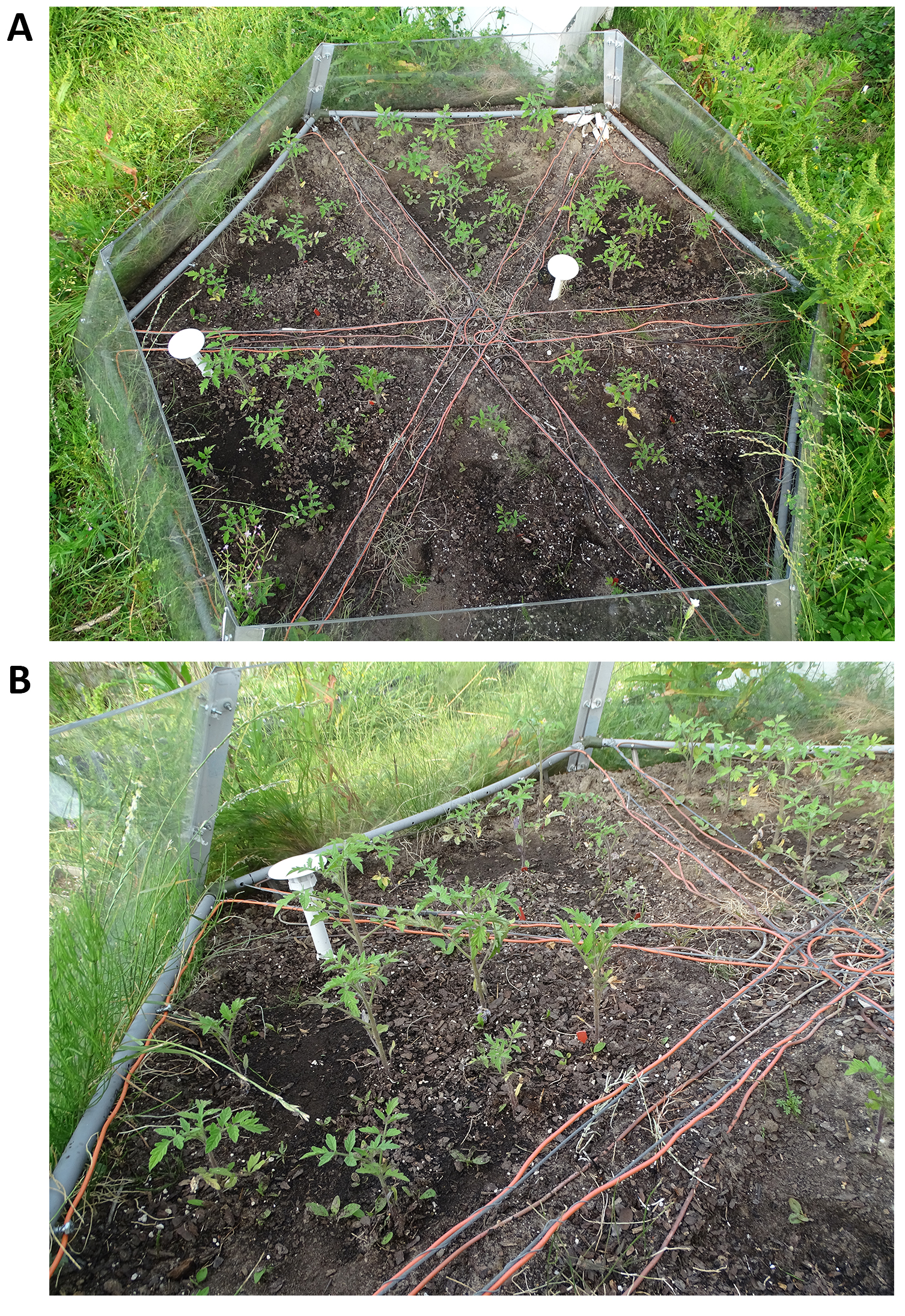

Supplement: Supplementary Figure 1 — Electronics layout within the weatherproofed plywood hutch, placed next to the OTCw+ (see Figure 3 ). Components are indicated by letters: (A) ESP8266 microcontroller, (B) Adalogger SD card data, (C) MOSFETs, (D) cooling fans, (E) 24V power supplies, (F) 230V sockets (right), (G) holes with fine mesh, (H) outdoor RCD powersocket, (I) fuses. [file SupplementaryFile1.zip › Supplementary Figure 9.JPEG]

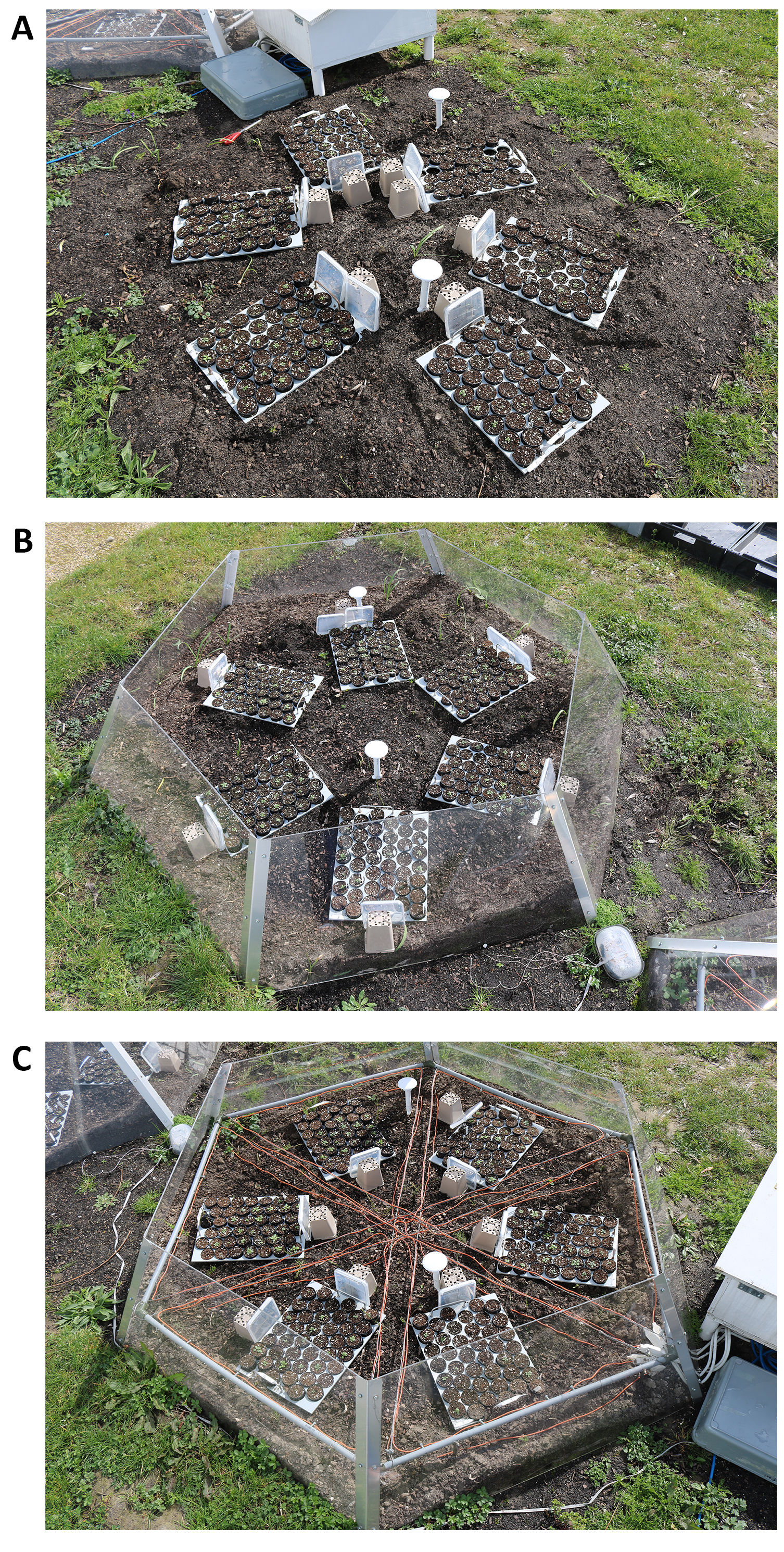

Supplement: Supplementary Figure 1 — Electronics layout within the weatherproofed plywood hutch, placed next to the OTCw+ (see Figure 3 ). Components are indicated by letters: (A) ESP8266 microcontroller, (B) Adalogger SD card data, (C) MOSFETs, (D) cooling fans, (E) 24V power supplies, (F) 230V sockets (right), (G) holes with fine mesh, (H) outdoor RCD powersocket, (I) fuses. [file SupplementaryFile1.zip › Supplementary Figure 8.JPEG]
